# Antibody‐Drug Conjugates to Treat Bacterial Biofilms via Targeting and Extracellular Drug Release

**DOI:** 10.1002/advs.202301340

**Published:** 2023-06-08

**Authors:** Anne Tvilum, Mikkel I. Johansen, Lærke N. Glud, Diana M. Ivarsen, Amanda B. Khamas, Sheiliza Carmali, Snehit Satish Mhatre, Ane B. Søgaard, Emma Faddy, Lisanne de Vor, Suzan H. M. Rooijakkers, Lars Østergaard, Nis P. Jørgensen, Rikke L. Meyer, Alexander N. Zelikin

**Affiliations:** ^1^ Department of Chemistry Aarhus University Aarhus C 8000 Denmark; ^2^ Department of Clinical Medicine Aarhus University Aarhus N 8200 Denmark; ^3^ Department of Infectious Diseases Aarhus University Hospital Aarhus N 8200 Denmark; ^4^ Interdisciplinary Nanoscience Centre (iNANO) Aarhus University Aarhus C 8000 Denmark; ^5^ Department of Medical Microbiology University Medical Center Utrecht Utrecht The Netherlands; ^6^ Department of Biology Aarhus University Aarhus C 8000 Denmark

**Keywords:** antibody‐drug‐conjugates, antimicrobials, biofilms, drug targeting

## Abstract

The treatment of implant‐associated bacterial infections and biofilms is an urgent medical need and a grand challenge because biofilms protect bacteria from the immune system and harbor antibiotic‐tolerant persister cells. This need is addressed herein through an engineering of antibody‐drug conjugates (ADCs) that contain an anti‐neoplastic drug mitomycin C, which is also a potent antimicrobial against biofilms. The ADCs designed herein release the conjugated drug without cell entry, via a novel mechanism of drug release which likely involves an interaction of ADC with the thiols on the bacterial cell surface. ADCs targeted toward bacteria are superior by the afforded antimicrobial effects compared to the non‐specific counterpart, in suspension and within biofilms, in vitro, and in an implant‐associated murine osteomyelitis model in vivo. The results are important in developing ADC for a new area of application with a significant translational potential, and in addressing an urgent medical need of designing a treatment of bacterial biofilms.

## Graphical abstract

Antibody‐drug conjugates are developed herein as a treatment against bacteria, planktonic, and within biofilms associated with therapeutic implants. The conjugate contains mitomycin C, an antimicrobial with a broad spectrum of activity. Conjugation to the *Staphylococcus aureus* specific antibody is via a disulfide linkage, for an extracellular drug release as the bacteria cell surface.

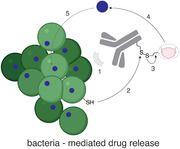

## 1 Introduction

Bacterial colonization of implanted biomaterials leads to infections that are a serious complication with a high socio‐economic and healthcare burden. Despite advances in surgery, infection remains a risk, with incidence rates of 1–2% for prosthetic knees and hips,^[^ ^1^ ^]^ 1–5% for prosthetic vascular grafts^[^ ^2^ ^]^ and up to 8.5% for spinal implants.^[^ ^3^ ^]^ Post‐operative prophylaxis often has little to no effect on the implant‐associated infections,^[^ ^4^ ^]^ and surgical intervention is often required to cure the patient.^[^ ^5^ ^]^ Patients ineligible for surgery are faced with either amputation of limbs or life‐long suppressive antibiotic therapy, which is also associated with significant morbidity.

The resilience of implant‐associated infections is linked to the formation of bacterial biofilms on and around the implant.^[^ ^5^, ^6^ ^]^ Bacteria in biofilms are embedded in a shared extracellular matrix, which offers protection from the immune system. Within the biofilm, slow‐growing or dormant sub‐populations emerge, and these populations are called “persister cells” because they survive extremely highconcentrations of all the antibiotics in current clinical use.^[^ ^7^ ^]^ Treatment of implant‐associated infections, therefore, remains a major healthcare challenge and requires a novel treatment paradigm.^[^ ^8^ ^]^

Novel therapies developed to specifically tackle biofilm infections often rely on the discovery of new antibiotics^[^ ^9^ ^]^ or the delivery of a high local dose of current antibiotics.^[^ ^10^ ^]^ Another approach to tackle bacterial infections is gaining momentum, based on the use of phages as a nature‐derived, bacteria‐specific treatment.^[^ ^11^ ^]^ Finally, with relevance to this work, antimicrobial measures can employ prodrug therapies where an inactive drug is circulating in the body and only activated at site of infection.^[^ ^12^ ^]^ In the latter case, promising results have very recently been obtained when prodrug activation was mediated by the host enzymes (typically proteases).^[^ ^13^ ^]^ In this study, we take advantage of drug repurposing and prodrug therapy to deliver an anti‐neoplastic drug that is highly effective against biofilms and will benefit from a prodrug therapy approach to minimize side effects. Specifically, we develop an antibody‐drug conjugate (ADC) of mitomycin C for the treatment of implant‐associated biofilm infections caused by *Staphylococcus aureus*, which is the most common culprit in prosthetic joint infections.^[^ ^6^ ^]^

The motivation for this endeavor lies in that ADCs are among the most successful tools of biomedicine, specifically for targeted drug delivery in cancer therapy.^[^ ^14^ ^]^ Within these prodrugs, the antibody arm is responsible for the association with a cognate cell surface ligand, judiciously chosen as a marker of a disease. In the majority of successful designs of ADCs, the linker between the antibody and the drug is designed to be stable during prodrug circulation in the blood and to be degraded upon cell entry.^[^ ^15^ ^]^ This measure ensures the highest specificity of action for an ADC against the nominated target. From the standpoint of chemistry, this is achieved using linkers that are stable in the oxidative environment of blood and neutral pH. Intracellular drug release is then achieved in response to acidification during the endosomal‐lysosomal trafficking of the ADC, using pH sensitive linkers between the protein and the drug. The significantly higher intracellular concentration of the thiol‐containing tripeptide glutathione (GSH) over its extracellular content makes disulfide linkages highly successful in the design of ADCs, in which case drug release occurs in the cell cytosol via thiol‐disulfide exchange. Finally, arguably the most successful linker methodology relies on peptide sequences that are degraded by the intracellular proteases such as cathepsin B.^[^ ^15^ ^]^ ADCs represent a highly successful paradigm with at least fourteen products approved for market,^[^ ^14^, ^16^ ^]^ all of which are developed for intracellular drug delivery and the treatment of cancer.

Examples of the use of ADC for the treatment of bacterial diseases (also termed antibody‐antibiotic conjugates, AAC) are scant.^[^ ^12^, ^17^ ^]^ In large part, this is because bacteria do not perform receptor‐mediated endocytosis, and methods should be designed to achieve drug release without the prodrug entry into the bacterial cells. In one successful example, an ADC has been developed to bind a bacterium and through opsonization to facilitate the uptake of the pathogen by the immune cells.^[^ ^18^ ^]^ Here, a rifamycin analog was conjugated to an antibody using a cathepsin‐sensitive linker such that drug release was mediated by mammalian proteases, within the immune cells.^[^ ^18^ ^]^ In another, very recent publication, O'Leary et al.^[^ ^13b^ ^]^ described an ADC that targets *Pseudomonas aeruginosa* and thus delivers a conjugated antimicrobial peptide to the bacterial cell surface, for drug release to be mediated by the host proteases. The latter work provides an early example that demonstrates that ADC can be developed into a powerful tool in the fight against bacterial pathogens, if drug release was engineered as an extracellular process. Indeed, extracellular drug release from a prodrug in the near‐vicinity of the targeted cell is a highly promising concept with applications in cancer treatment.^[^ ^19^ ^]^ Triggered extracellular drug release can be engineered with the knowledge of the enzymatic fingerprint of a disease^[^ ^20^ ^]^ or using an externally added chemical stimuli.^[^ ^21^ ^]^ This approach to targeted drug delivery can be particularly well suited in the design of ADCs toward the treatment of the implant associated bacterial infections. An antibody can be selected to anchor the prodrug to the surface of the bacteria within the biofilm before triggering release at the bacterial cell surface, using an external small molecule or via an interaction with the specific components of the bacterial cell surface and/or biofilm. This approach potentially opens the door to the use of the most potent antimicrobials that are effective against biofilm infections, but have not been implemented in the clinic due to their side effects. Local drug release within the biofilm will minimize the systemic concentration of active drug while maximizing its therapeutic impact.

Small molecule‐triggered therapeutic activity has been applied to on‐demand activation of the chimeric antigen receptor T cells,^[^ ^22^ ^]^ to dissolve implanted hydrogels and thus to release the second dose in a prime‐boost vaccination approach,^[^ ^23^ ^]^ as well as for drug release from the “click‐to‐release” linkers.^[^ ^21^ ^]^ These elegant techniques rely on safe molecules, possibly marketed as therapeutics, to act as a switch in a molecularly programmed gate. We hypothesized that a marketed mucolytic agent, *N*‐acetyl cysteine, can act as such a switch to trigger drug release from the ADCs that in their structure feature a disulfide‐linkage between the antibody and the drug.

From a different perspective, we also considered that cell surfaces, mammalian^[^ ^24^ ^]^ and bacterial,^[^ ^25^ ^]^ are often characterized by abundant accessible thiols. For mammalian cells, these thiols have been used to initiate drug release and to achieve cell entry, through conjugation to the cell surface thiols via thiol‐disulfide exchange and subsequent internalization of the cell‐bound cargo.^[^ ^24^, ^26^ ^]^ To the best of our knowledge, this technique has not been applied to bacterial cells, though the presence of thiols on the surface of bacterial outer membrane (for the Gram‐negative bacteria) or the cell wall (for Gram‐positive pathogens) has been experimentally confirmed in a number of studies.^[^ ^25^ ^]^ It therefore seemed highly plausible that these surface thiols may be suited to initiate drug release via thiol‐disulfide exchange.

**Figure 1:**
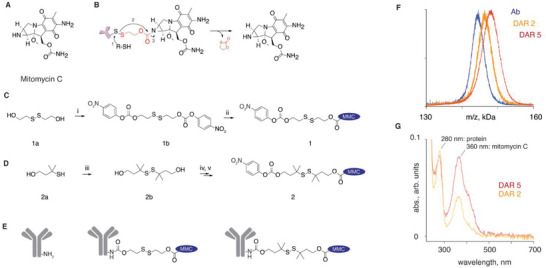
A) Chemical formula of mitomycin C; B) Schematic illustration of the use of self‐immolative linker to achieve release of the thiol‐free drug, mitomycin, from its prodrugs (e.g., antibody‐drug conjugates) triggered via thiol‐disulfide exchange; C–E) Schematic illustration of syntheses of the amine‐reactive, mitomycin C containing linker‐drug conjugates **1** and **2**, for a single step conjugation to antibodies. Experimental conditions: i) **1a** (1 equiv.), 4‐nitrophenyl chloroformate (2.2 equiv.), TEA (4 equiv.), CH_2_Cl_2_, 0 °C to r.t., 2 h, 61%; ii) **1b** (2.4 equiv.), mitomycin C (1 equiv.), TEA (1.5 equiv.), HOBt (6.7 equiv.), DMF, 24 h, 57%; iii) **2a** (1 equiv.), CuCl_2_ (5 equiv.), CH_2_Cl_2_:EtOAc (1:1), HCl (0.5 m), 1.5 h, r.t., 73%; iv) **2b** (1 equiv.), 4‐nitrophenyl chloroformate (2.8 equiv.), TEA (2.5 equiv.), CH_2_Cl_2_, 3 h, r.t., 69%; v) **2c** (1 equiv.), mitomycin C (1 equiv.), TEA (1.1 equiv.), HOBt (1.5 equiv.), CH_2_Cl_2_, 0 °C to r.t. over 10 min., r.t. for 24 h, 25%; conjugation to antibodies: Ab (c_protein_ > 3 g L^−1^), **1** or **2** (55 equiv.), 10 mm PBS, pH 7.4, 2 h; Characterization of the ADC with drug‐to‐antibody (DAR) ratio of 2 and 5 using F) MALDI and G) UV–vis spectroscopy.

The above‐presented design considerations dictated the final composition of ADCs engineered in this work. We used commercially available antibodies against *S. aureus* and synthesized ADCs with a disulfide linkage between the antibody and the drug mitomycin C (**Figure** **1**A). Mitomycin C was chosen due to its outstanding antimicrobial activity against biofilms and the persister cells within.^[^ ^27^ ^]^ Toward the overall goal, we i) established the mitomycin C containing ADC and validated drug release from these prodrugs in response to *N*‐acetyl cysteine (NAC) and a panel of other biologically relevant thiols; ii) visualized specific interaction of *S. aureus‐*specific ADCs with planktonic bacteria and biofilms, iii) quantified the antibacterial efficacy of ADCs against planktonic bacteria and biofilms, and iv) demonstrated in vivo therapeutic effects of the ADCs in a murine implant‐associated osteomyelitis model. Unexpectedly, we established that drug release is independent of NAC in the presence of bacteria, and we attribute the bacteria‐triggered drug release to interaction of the ADCs with thiols on the bacterial cell surface. The importance of our data is in that we re‐engineer the highly successful therapeutic tool, ADC, to the previously un‐explored area of use, and establish a novel modality of treatment against condition with a high medical need. We anticipate that these data will have a strong academic impact and believe that the ADCs have a promising translational perspective.

## 2 Results

### 2.1 Design of ADC

Disulfide chemistry is among the most investigated linker technologies in drug delivery. The one limitation commonly encountered in these endeavors is that a disulfide is a bond between two sulfur atoms, and only few marketed drugs feature thiols in their structure. A successful route to overcome this limitation and apply disulfide chemistry to a wide range of drugs is the use of “self‐immolative linkers.”^[^ ^28^ ^]^ These linkers are designed to undergo fast decomposition upon a scission of the disulfide bond, most commonly via intramolecular cyclization, to afford a traceless release of the drug from its conjugate in its pristine form (Figure 1B). Mercaptoethanol dimer (compound **1a,** Figure 1C) is a convenient, readily available starting material for these syntheses. In our work, **1a** was converted into a symmetrical homobifunctional amine‐reactive carbonate, and subsequently reacted with mitomycin C to afford a drug‐linker conjugate (**1**) for a single‐step conjugation to an antibody. Literature survey reveals that stability of disulfides against decomposition in blood significantly improves when carbon atom(s) adjacent to sulfurs are substituted with, for example, methyl groups, which create steric shields to non‐specific exchange between this disulfide and cysteine thiols on albumin and other serum proteins. Learning from this, linker **2** was designed, starting with 3‐methyl‐3‐sulfanylbutan‐1‐ol (compound **2a,** Figure 1D) which was oxidized using CuCl_2_ into the sterically hindered disulfide (**2b**). This was reacted with 4‐nitrophenyl chloroformate to afford the homo‐bifunctional carbonate **2b** and then with mitomycin C to obtain the final drug‐linker conjugate **2**.

Compounds **1** and **2** are amine‐reactive and were used to conjugate to the antibodies in their aqueous solutions (Figure 1E). ADCs were purified via gel filtration and characterized for composition using matrix assisted laser desorption ionization mass spectrometry (MALDI), which revealed that protein conjugates had molar mass higher than the pristine antibody and allowed to calculate the drug‐to‐antibody ratio (DAR) for the conjugates (results for ADC based on linker **1**, DAR = 2 and 5 are shown as examples in Figure 1F. UV–vis spectra of the ADC also revealed signature absorbance for both, the protein and the conjugated drug, with absorbance for the latter expectedly increasing with DAR (Figure 1G). Finally, size exclusion chromatography and gel electrophoresis confirmed that ADC with DAR as high as 8–13 show minimal aggregation in solution upon storage (Figure S1, Supporting Information).

### 2.2 Drug Release

**Figure 2:**
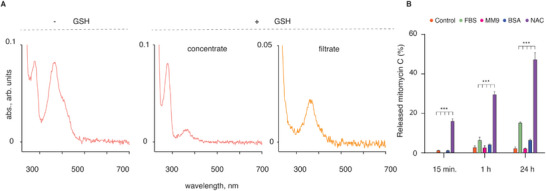
A) Qualitative investigation of drug release from ADC using UV–vis spectroscopy showing the spectrum of the ADC (‐GSH) and the spectra after the treatment with GSH and separation of the mixture by gel filtration, separately for the concentrate (the protein) and the filtrate (the drug); B) Drug release from ADC in PBS containing NAC (5 mm), bovine serum albumin (BSA, 0.76 µm), bacterial culture medium MM9, or FBS (10% in RPM1‐1640 cell culture medium), over 24 h of incubation; control = PBS only; results are based on three independent experiments and shown as mean ± SD; statistical significance determined using two‐way ANOVA analysis; for brevity, only selected significance values are presented, ***: *p* < 0.001.

To investigate drug release, ADCs were treated with a natural reducing agent, a thiol‐containing tripeptide GSH (5 mm). The resulting mixture was separated by size via spin filtration and then the filtrate and the concentrate were independently analyzed via UV–vis spectroscopy (**Figure** **2**A). The concentrate (high molar mass) solute volume revealed the presence of the protein with minor residual content of the drug, while the filtrate (low molar mass) exhibited the UV–vis signature of mitomycin C without the protein. These data validate the success in the design of ADC that releases their conjugated cargo via thiol‐disulfide exchange.

Quantitative analysis of drug release from ADC was performed using high‐performance liquid chromatography (HPLC, Figure 2B). To this end, the ADC was incubated in phosphate buffered saline (PBS) in the presence of NAC (5 mm), albumin (0.76 µm), or fetal bovine serum (10% in RPM1‐1640 cell culture medium), as well as in MM9 bacterial cell culture medium which was later to be used in antimicrobial testing. The common biochemical reducing agent dithiothreitol (DTT, 5 mm) was used as a positive control to achieve complete drug release from ADC. During the initial 15 min of incubation, ADC based on linker **1** exhibited negligible drug release in all conditions except for a treatment with NAC. With longer incubation, drug release became noticeable from ADC incubated in the presence of FBS, reaching ≈20% after 24 h. Nevertheless, the release triggered by NAC was substantially higher at all the time points. For the ADC designed using linker **2**, no drug was released upon incubation of ADC for 24 h in FBS or in PBS with BSA or NAC (data not shown). The only reducing agent that released mitomycin C was DTT, which has limited if any potential of use in vivo. Based on these results, in all subsequent experiments, we used ADC based on linker **1**.

### 2.3 ADCs Associate with the Surface of *S. aureus* and Retain Immune‐Activating Properties

**Figure 3:**
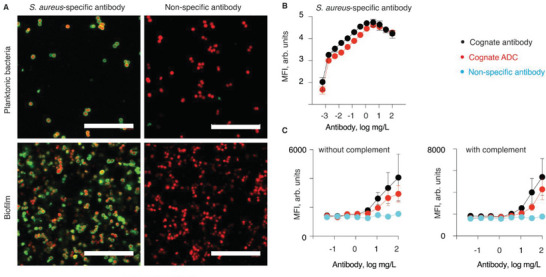
A) CLSM images of *S. aureus* (red) following incubation with Alexa467‐labeled *S. aureus*‐specific antibody from rabbits or IgG_1_ specific to fluorescein isothiocyanate (anti‐FITC IgG1) (green). Bacterial cells were visualized either by SYTO41 staining (biofilm) or GFP fluorescence (planktonic cells). Scale bars = 20 µm. B) Comparison of ADC (red) and the parent antibody (black) in their ability to bind to *S. aureus* (strain Newman‐spa/sbi‐KO‐mAm), quantified by flow cytometry as fluorescence from the bound secondary antibody; C) Specific/non‐specific (black or blue, respectively) antibody‐ or ADC‐mediated phagocytosis of *S. aureus* by human neutrophils over 15 min, quantified by flow cytometry measuring the GeoMFI of neutrophils with mAmetrine‐labeled *S. aureus* (strain Newman Δspa/Δsbi_mAm). Each data point represents mean ± SD of n = 3 separate experiments. Panels (B,C): MFI = Mean fluorescence intensity.

Next, we aimed to validate antibody binding to bacteria. The optimal antibody for a design of ADC to deliver drugs to bacteria should bind to bacterial cells when growing planktonically and as biofilm, despite the differences in the expression of surface proteins in these two growth modes. To investigate this, we fluorescently labeled commercial polyclonal *S. aureus*‐specific antibody from rabbits (specific antibody) and visualized bound antibodies by confocal laser scanning microscopy (CLSM, **Figure** **3**A). As a control, we also labeled IgG_1_ specific to fluorescein isothiocyanate (anti‐FITC IgG1) to evaluate unspecific binding by off‐target antibodies to *S. aureus* cells, which could happen by interaction between the antibody's Fc region and Protein A on the bacterial surface. Both planktonic cells and biofilms bound the nominally cognate antibody, while the off‐target antibody was not detected (Figure 3A). The specific antibody was thus a suitable vehicle for delivering antimicrobials to *S. aureus* in suspension and within biofilms.

Conversion of an antibody into the corresponding ADC may affect cognate interactions of the protein at both Fab (antigen recognition) and Fc (secondary interactions) regions. To test this, we first compared the *S. aureus*‐binding properties of the parent antibody and the ADC constructed therefrom (DAR 8, Figure 3B). Antibodies or ADCs were incubated with *S. aureus* and thereafter fluorescently conjugated secondary antibody was added for the detection of bound protein. The parent antibody exhibited superior bacterial binding compared to the ADCs at the lowest tested concentrations (0.045–0.4 mg L^−1^; p<0.009, two‐way ANOVA). However, this was not the case at antibody concentrations used in the subsequent antimicrobial assays of this study (vide infra). The results of this experiment complement visual observations via CLSM (Figure 3A) and illustrate that the antibody and the ADC successfully bind the bacteria via cognate interactions.

From a different perspective, antibodies and ADC can also help to eliminate bacterial pathogens through opsonization, that is, binding to bacterium, possibly attracting complement proteins, and in doing so facilitating phagocytosis by neutrophils.^[^ ^18^ ^]^ This mode of action relies on the recognition between the Fc part of the antibody/ADC by the neutrophils and/or by the complement proteins, and this cognate interaction can possibly be altered by design of ADC. To investigate this, we used the Newman Δspa/Δsbi_mAm strain of *S. aureus* which expresses the fluorescent protein mAmetrine. Bacteria were incubated together with ADCs (specific and non‐specific) or native antibodies, with or without 1% IgG/IgM depleted pooled human serum as complement source.^[^ ^29^ ^]^ Following this, the bacteria were incubated with human neutrophils, and the phagocytosis activity was quantified via flow cytometry (Figure 3C). In the absence of complement proteins, *S. aureus*‐specific antibody and the ADC derived thereof promoted bacterial internalization by the neutrophils. In contrast, bacteria incubated with the non‐specific antibody exhibited little, if any, internalization by the neutrophils. These data illustrate that both the cognate antibody and the cognate ADC bind to bacteria and facilitate internalization by neutrophils. The difference between the parent antibody and the ADC was statistically significant only at the highest protein concentration (100 mg L^−1^) but not at the lower concentrations. Interestingly, addition of complement proteins afforded little change in the levels of bacteria internalization. This suggests that the cognate antibody and the ADC mediate phagocytosis via Fc gamma receptors. Together, results in Figure 3 illustrate that modification of the antibody with mitomycin C (at least up to a DAR of 8) had little effect on the cognate interactions via the Fab fragment with bacteria or via the Fc fragment with the neutrophils, which is highly important for the utility of the ADC as antimicrobial agents and potentially also as immune‐stimulating agents.

### 2.4 Antimicrobial Efficacy of Mitomycin C

We chose mitomycin C as our antimicrobial agent due to its exceptional antimicrobial effect against biofilms, which otherwise have a high tolerance to antibiotics. To assess the concentration of mitomycin C needed to inhibit or kill *S. aureus*, we determined the minimum inhibitory concentration (MIC), the minimal biocidal concentration (MBC) required to kill > 99.9% of a planktonic culture, and the minimum biofilm eradication concentration (MBEC) required to fully eradicate viable cells from a biofilm (**Table** **1**). The efficacy of antimicrobials differs slightly in various buffers and media, and we, therefore, determined the antimicrobial efficacy in all the solutions relevant for the subsequent antimicrobial testing. The similarity between MBC and MBEC values confirms that the antimicrobial efficacy of mitomycin C is not impacted by biofilm formation. Noteworthy, these values appear to be significantly lower than the reported values of the plasma level of mitomycin C that is reached in cancer therapy.^[^ ^30^ ^]^

**Table 1:** The antimicrobial efficacy of mitomycin C against *S. aureus*

Media	MIC [mg L^−1^]	MBC [mg L^−1^]	MBEC [mg L^−1^]
BHI	0.4	0.4	1–4
MM9 media	0.4	0.4	1–2
MM9 buffer	–	0.1	1–8
MM9 buffer + NAC	–	0.1	1–4

### 2.5 Antimicrobial Efficacy of ADCs

**Figure 4:**
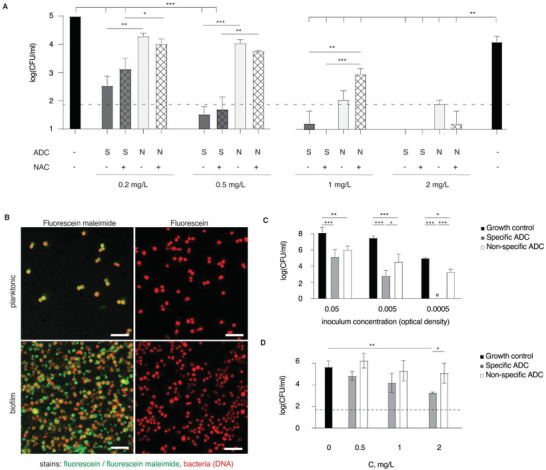
A) Quantification of live planktonic *S. aureus* in colony forming units after treatment with ADC and optionally with NAC; B) Visualization of the surface accessible thiols on *S. aureus* (labeled with SYTO60, red) in planktonic state or within a biofilm using fluorescein maleimide or fluorescein (green) as a control. S scale bars = 10 µm; C) Antimicrobial activity of the ADC (0.5 µg mL^−1^) against planktonic bacteria, comparing the ADC cognate to *S. aureus* (“specific ADC”) or to FITC (“non‐specific ADC”). Y axis shows viable *S. aureus* after 2 h incubation of ADC with different starting concentrations of bacteria. D) Antimicrobial activity of the ADC against the *S. aureus* biofilms after 15 min incubation with ADCs to allow binding followed by 2 h incubation to allow ADCs to take effect before harvesting bacteria for CFU enumeration. Concentrations are expressed in equivalent concentration of mitomycin C. In panels (A,C,D), results are based on three biological replicates and presented as mean ± SD; statistical evaluation was conducted via a two‐way ANOVA using log‐transformed values of CFU count; for brevity, only selected statistical significance is presented; * *p* < 0.05; ** *p* < 0.01; *** *p* < 0.001.

The designed ADCs were tested as agents for delivery of antimicrobials to bacteria in suspension. To this end, *S. aureus* was incubated with the ADC for 2 h, and then diluted and plated on solid media to quantify viable cells as colony forming units (CFU). The ADC samples used in these experiments were designed using either specific pIgG (typical DAR = 7–8) or non‐specific anti‐FITC IgG1 (typical DAR = 13) and tested in a range of concentrations from 0.2 to 2 mg L^−1^ (equivalent mitomycin C concentration). Incubation of ADC with bacteria was performed in the presence or absence of NAC to trigger the release of mitomycin C. At the highest concentration tested (2 mg L^−1^), incubation with both specific or non‐specific ADC, with or without added NAC, decreased the number of viable bacteria to a value below the detection limit, likely indicating a non‐specific drug release from all ADCs, even in the absence of NAC (**Figure** **4**A). At lower concentrations, best seen at 0.5 mg L^−1^, the antimicrobial activity of specific and non‐specific ADCs was significantly different, and the cognate ADC decreased bacterial cell counts to below the detection level, whereas the non‐specific ADC only afforded a minor decrease. These data illustrate the highly desired outcome, namely that cognate ADCs exhibit pronounced therapeutic efficacy of antimicrobials at low concentrations by targeted delivery to the cell surface.

The results in Figure 4A also demonstrate that NAC had no effect on the antibacterial activity of ADCs. This observation is highly unexpected. As such, it does not rule out that a NAC‐mediated drug release occurred (as shown in Figure 1), but it indicates that the bacterial killing shown in Figure 4A is due to a drug release mechanism that is NAC‐independent. One plausible mechanism can involve the thiol groups nested at the cell surface. For mammalian cells, exofacial sulfhydryls have become a highly important player in drug delivery at cell surfaces^[^ ^31^ ^]^ and/or to the cell interior.^[^ ^24^, ^26^ ^]^ Thiols are also abundant on bacterial cells,^[^ ^25^ ^]^ making it possible that interaction of ADCs with the cell surface ensues a thiol‐disulfide exchange and release of the active drug. We therefore visualized thiol groups on the surface of *S. aureus* via fluorescent labeling. Bacteria were exposed to a thiol‐reactive fluorescein maleimide or its non‐reactive parent compound, fluorescein, and imaged by CLSM (Figure 4B). Exposure to fluorescein maleimide produced a strong fluorescent signal associated with the surface of bacteria, whereas no fluorescence was observed in the samples incubated with fluorescein. These data indicate that the *S. aureus* cell surface is rich in accessible, reactive thiol groups.

In designing the next experiment, we considered that the interaction between bacteria and ADC, both specific and non‐specific, is concentration‐dependent, and that antimicrobial efficacy of ADC should therefore be dependent on the concentration of bacteria in suspension. Indeed, in our hands, the antimicrobial activity of the ADC was strongly dependent on the bacterial cell concentration (Figure 4C). Moreover, the bacterial cell concentration was highly important in defining the relative efficacy of antimicrobial activity between the cognate ADC and its non‐specific counterpart. At the highest cell concentration, bacterial cell killing was strong for both specific and non‐specific ADC preparations, and the difference between the two ADCs was not significant. In contrast, at the lowest bacterial cell concentration, the efficacy of treatment with the cognate ADC was significantly higher than that for the non‐specific ADC. This experimental finding is readily explained by the difference in potency between the two ADCs. The low‐potency non‐specific interactions may be significant at a high cell concentration, but they should become less significant at the low cell concentrations. At the same time, the high potency cognate interactions remain pronounced within the studied range of cell concentrations, and this leads to an increasing difference between specific and non‐specific ADC by efficacy of antimicrobial activity.

Having established the antimicrobial effect of ADCs against planktonic *S. aureus*, we also aimed to validate if this mode of drug delivery is effective against the bacterial biofilms (Figure 4D). The antimicrobial effect on biofilms was concentration‐dependent, and at 2 mg L^−1^ concentration of ADC, the antimicrobial effect of specific ADCs was significantly higher than the non‐specific ADCs. The biofilm was not eliminated during the treatment, but it should be stressed that the treatment time was very short. Biofilms were only exposed to ADCs in solution for 15 min and then incubated for 2 h after unbound ADC was removed. Even after this short incubation time, the number of viable bacteria decreased by more than 100‐fold, illustrating that ADCs are efficacious against *S. aureus* biofilms.

### 2.6 Therapeutic Efficacy of ADCs against Biofilm Infections

**Figure 5:**
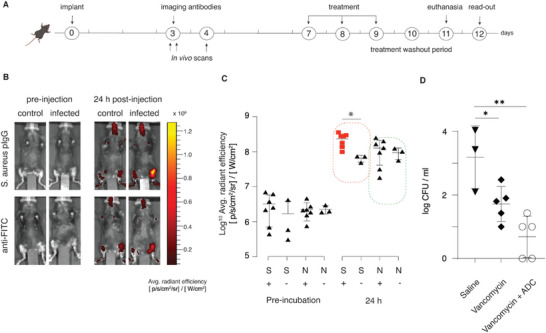
Pilot imaging and therapy in vivo study. A) schematic presentation of the experiment timeline; B) In vivo imaging of deposition of the fluorescently labeled antibodies, cognate to *S. aureus* or to fluorescein, in a limb with a sterile implant or in the limb with an infected implant (osteomyelitis model); C) Quantification of deposition of the fluorescently labeled antibodies; S = *S. aureus* specific antibody; N = non‐specific antibody; in both cases labeled with Alexa Fluor NHS ester 687; “+” signifies animals with an infected implant, “‐ “ is for animals with a sterile implant; D) Antimicrobial effects in mice upon treatment with vancomycin, ADC (cognate to *S. aureus*), or the combination of two agents. In panels (C,D), each data point represents radiant efficiency from C) implanted leg or represents D) a single animal. Statistical evaluation was conducted via two‐way ANOVA (panel (C)) or one‐way ANOVA (panel (D)).

In vivo evaluation of ADC was performed in 8–10 weeks old C57bl/6j mice, in an implant‐associated osteomyelitis model (**Figure** **5**A).^[^ ^32^ ^]^ Briefly, stainless steel insect pins were surgically inserted into the tibia after they had been inoculated in an overnight culture of *S. aureus* SAU060112 (ref. [33]). Control mice received a sterile implant. On day 3 after surgery, mice were administered with fluorescently labeled antibodies to verify the ability of the cognate antibody to accumulate at the site of infection. For comparison, fluorescently labeled anti‐FITC antibodies were administered to evaluate non‐specific accumulation of antibodies. In vivo full body imaging was used to visualize and quantify fluorescence from antibodies in the mice with sterile compared to infected implants, and from the specific antibodies compared to non‐specific antibodies (Figure 5B,C). 24 h after antibody administration, fluorescence from *S. aureus*‐specific antibodies was higher from infected implants compared to sterile implants (marked in Figure 5C with “+” and “‐” respectively), whereas the signal for the non‐specific antibody was the same from the infected and the sterile implants. These data indicate that the *S. aureus*‐specific antibody bound to and accumulated at the site of infection, as is required for the desired site‐specific therapeutic activity.

7 days after the surgery, animals with infected implants were randomly divided into three treatment groups that were administered with saline, vancomycin as a monotherapy, or vancomycin combination therapy with ADC, for 3 consecutive days. This pilot experiment was designed specifically, such that the two treatment arms contained vancomycin, which is first line treatment of MRSA infection.^[^ ^34^ ^]^ The administered dose of vancomycin was 110 mg kg^−1^/12 h/s.c. For the ADC (DAR 7–8), the administered dose was 5 mg kg^−1^/24 h/i.v. Here and in all in vivo experiments, ADC concentration is expressed in total solids content (not equivalent concentration of mitomycin C); 5 mg kg^−1^ ADC corresponds to ≈4.9 mg kg^−1^ of the antibody and 86 µg kg^−1^ of mitomycin C. Treatments were administered on days 7, 8, and 9 and followed by a 2‐day washout period before animals were euthanized, and the bacterial load was quantified by CFU enumeration. Quantification of bacteria on the surface of the recovered implant revealed that vancomycin alone led to an approximately tenfold decrease in the bacterial load (Figure 5D) while addition of ADC to the vancomycin treatment led to an ≈100‐fold reduction, although with a limited sample size the effect was not statistically significant. Nevertheless, this pilot study was important in that it revealed that the ADC treatment did not have any detrimental effect on animal well‐being, and it also provided the first indication of therapeutic efficacy of the ADC in vivo.

**Figure 6:**
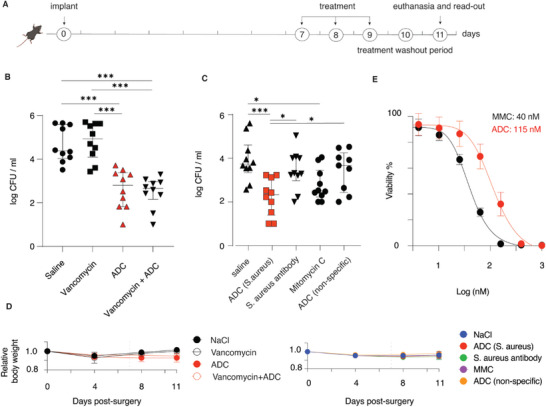
A) Schematic representation of the workflow timeline for the quantification of antibacterial effects in vivo; B) Experimental antimicrobial effects upon treatment with vancomycin (110 mg kg^−1^/12 h/s.c.), ADC (5 mg kg^−1^/24 h/i.v.), or the combination of the two agents. C) Experimental antimicrobial effects upon treatment with ADC (cognate to *S. aureus* or fluorescein, in both cases 5 mg kg^−1^/24 h/i.v.), with the anti‐*S. aureus* antibody (5 mg kg^−1^/24 h) or mitomycin (86 µg kg^−1^/24 h/i.v.) taken individually. In panels (B,C), each data point represents an individual mouse; statistical significance was calculated via a one‐way ANOVA using the log‐transformed experimental CFU values. D) Relative body weight for mice that underwent treatments as presented in panels (B) and (C); E) In vitro cytotoxicity of mitomycin and an ADC derived thereof for MOLT‐4 cells (a human T lymphoblast cell line) following a 72 h incubation (presented results are based on three independent experiments and presented as mean ± SD) *p ≤ 0.05; *** *p* ≤ 0.001.

Next, we performed two experiments to investigate the therapeutic potential of the ADC. First, ADC mono‐therapy was compared to mono‐therapy with vancomycin or a combination therapy with vancomycin and ADC (**Figure** **6**A,B). In this experiment, unlike the pilot study discussed above in Figure 5, vancomycin treatment alone afforded no effect on the bacterial burden, which is explained by the differences in the implant inoculation and the implant recovery protocols employed in the two experiments. In stark contrast, ADC treatment was highly effective and statistically significant compared to saline treatment and the vancomycin treatment arms. Concurrent administration of vancomycin and the ADC afforded no added benefit for the therapeutic outcome. These data provide a strong indication of efficacy of treatment with the developed ADC in vivo, even after a very short (relative to the clinically standard, ref. [35]) treatment period of three single doses of the ADC.

Last, the treatment efficacy of was quantified side by side for the specific and non‐specific ADC based on antibodies cognate to *S. aureus* or off‐target antibodies, and also to the cognate antibody or mitomycin C taken individually (Figure 6C). The most important observation from this experiment is that the therapeutic benefit of the specific ADC cognate to *S. aureus* was statistically significant compared to the treatment with saline, to the treatment with the unconjugated antibodies, and to the treatment with non‐specific ADC. The superior therapeutic effect of the *S. aureus*‐specific ADC supports our presumption that the bacteria are killed by mitomycin C released from the ADCs upon interaction with the biofilm. Mitomycin C monotherapy had also a statistically significant effect, which is consistent with the antibacterial efficacy that we and others have observed for the drug in bacterial cell culture.^[^ ^27^, ^36^ ^]^ Importantly, the mice exhibited a minor change in the body weight due to surgery but not due to treatment (Figure 6D), indicating that the treatment was well tolerated. While the current data does not reveal statistical significance between the treatment with ADC and mitomycin, we note that the significance for the mitomycin treatment compared to saline has a *p*‐value of 0.038, whereas, for the treatment with ADC, the *p*‐value is 0.0004. It illustrates that statistical confidence in the treatment by ADC is almost a hundred times higher than for the treatment with mitomycin. Future studies with larger groups of animals and optimized dosing may better reveal the therapeutic benefit of the ADC. As it stands, we illustrate that the efficacy of treatment with ADC is at the very least on par with pure mitomycin C. At the same time, in vitro cell culture experiments illustrate that ADC is less toxic when compared to mitomycin C (Figure 6E), which implies that the ADC treatment may be associated with fewer side effects.

## 3 Discussion

In this study, we developed an ADC that targets *S. aureus* and exhibits antimicrobial activity in vitro and in vivo. To our knowledge, this is the first study to develop and demonstrate the potential for using ADCs in antimicrobial therapy directed at extracellular infections and biofilms. Only a few prior studies have developed ADCs for antimicrobial therapies,^[^ ^17^ ^]^ and the focus was mainly been on intracellular drug release, to kill bacteria internalized by immune cells.^[^ ^18^, ^37^ ^]^ Effective antimicrobial therapies against biofilm infections typically require a high load of antibiotics and lengthy treatment.^[^ ^38^ ^]^ Enhanced deliverable payload can be achieved using tools of nanomedicine, such as liposomes or solid nanoparticles,^[^ ^10^, ^39^ ^]^ whereas enzymes and nanozymes may prove useful for localized drug synthesis schemes.^[^ ^12^, ^40^ ^]^ Each of these techniques has its own merits. ADCs have a competitive edge in that multiple products are already on the market and numerous candidates navigate through clinical trials, illustrating that technology for the production of ADC is well‐established, and clinical acceptance of these agents is very high.^[^ ^14^ ^]^ However, as described to date, ADC focus on cancer treatment and intracellular drug release.^[^ ^14^ ^]^ For the treatment of bacterial pathogens and specifically biofilms, a novel mode of action for ADC is required. Our initial thought was to use ADC for drug targeting and thereafter achieve a localized drug release using an independently administered trigger for drug release. Somewhat surprisingly, we observed that this trigger was not required and ADC proved to be potent and efficacious as a monotherapy, in vitro and in vivo.

The ADCs synthesized in this work release the payload via disulfide reshuffling. We validated via thiol staining that the bacterial cell surface has abundant thiols, which strongly suggests that bacteria are competent to participate in the thiol‐disulfide exchange, to initiate the drug release from the ADC. Indeed, disulfide reshuffling has been documented at the surface of mammalian cells in numerous studies.^[^ ^24^, ^26^ ^]^ For bacteria, such reports are scant and in fact, antibiotics acting in the cytoplasm were inactivated via their conversion to disulfide‐containing derivatives, possibly suggesting conjugation to the bacterial cell surface and thereby arrested drug cell entry.^[^ ^25a^ ^]^ In recent studies, Shchelik and Gademann^[^ ^41^ ^]^ observed that the disulfide containing derivatives of vancomycin and cephalosporin were superior to the parent antibiotic molecules, although no mechanism for the involvement of the disulfide functionality was provided to explain the enhanced drug efficacy of the newly synthesized compounds. In our hands, attempts to block the bacterial cell surface thiols with maleimide‐based sulfhydryl poisons^[^ ^24c^ ^]^ were met with limited success (data not shown). Thus, detailed understanding of the mechanism of activity of the ADC and the role of bacteria‐mediated drug release requires significant further experimentation. Nevertheless, all the data collected in this work point toward the bacteria‐mediated drug release because i) spontaneous drug release from the ADC in the MM9 culture media was insignificant; ii) external triggering of drug release by added NAC did not enhance antimicrobial activity; and iii) cognate ADC exhibits superior efficacy and potency compared to the non‐specific counterpart.

We believe that the results of this study are highly important in that they open doors for using mitomycin C as an antimicrobial agent. Mitomycin C works by crosslinking DNA and is thus highly cytotoxic. It is a highly attractive agent for the use as an antimicrobial because it is equally effective against actively growing cells (susceptible to conventional antibiotics) and slow‐growing or dormant persister cells (tolerant to conventional antibiotics). Indeed, a number of studies have confirmed the potential of mitomycin C as a potent antimicrobial against biofilms due to its effect on persister cells.^[^ ^27^, ^36^, ^42^ ^]^ Our study illustrates that mitomycin may be formulated as a safer, targeted prodrug, for a localized drug release within the biofilm.

One limitation of this study is that the presented in vivo data do not present superiority of ADC over mitomycin as an antimicrobial therapy. To achieve this, a study into the maximum tolerated dose for the ADC and the parent drug is being set, during which we will also gain further insight into the toxicity of treatment with ADC. Another limitation is that the ADC designed in this work targets only *S. aureus*, whereas the bacterial biofilms often include multiple bacterial species.^[^ ^35^, ^43^ ^]^ Nevertheless, it is conceivable that the *S. aureus*‐specific antibody can deliver and release mitomycin in polymicrobial biofilms where the antimicrobial activity will impact *S. aureus* as well as other bacteria in the biofilm, via the bystander toxicity effect. The targeting mechanism thus may not need to be tailored toward every single pathogen, but only to one of the abundant organisms in a polymicrobial infection. We confirmed that as an antimicrobial agent, mitomycin C is active against Gram positive and Gram negative bacteria alike, as well as strains that are resistant to conventional antibiotics (**Table** **2**). Investigation of the ADC efficacy against polymicrobial and antibiotic‐resistant biofilms is currently underway and will be reported the upcoming publications.

**Table 2:** The antimicrobial efficacy of mitomycin C against different pathogens

Pathogen	MIC [mg L^−1^]	MBC [mg L^−1^]
*Escherichia coli*	1–2	2
*Staphylococcus epidermidis*	0.5–1	1
*Pseudomonas aeruginosa*	1–2	4
*Enterococcus faecalis*	2	4
Methicillin‐resistant *Staphylococcus aureus*	0.25	0.25–0.5

## 4 Conclusions

ADCs, one of the most successful platforms for targeted intracellular drug delivery, are developed in this study toward a new area of applications, specifically targeted delivery of drugs to treat bacterial biofilms. Our results point to a novel mechanism for drug release at the bacterial cell surface, namely via a thiol‐disulfide reshuffling between the disulfide‐conjugated drug and the exofacial cellular thiols. We demonstrate antimicrobial efficacy of the ADC in vitro and in vivo in an implant‐associated osteomyelitis model. Treatment of biofilms is a tremendous socio‐economic burden and represents an unmet medical need. We believe that our findings open up significant opportunities to the treatment of bacterial biofilms, with a potential to identify ligands for optimized targeting and/or therapeutic molecules for enhanced antimicrobial effects.

## 5 Experimental Section

### General Information

All chemicals and reagents were purchased from commercial vendors (Sigma Aldrich, Tokyo Chemical Industry, Selleck chemicals) and used without further purification, unless otherwise stated. Triethylamine (TEA) and *N,N*‐dimethylformamide (DMF) was purchased anhydrous and the deuterated solvents were purchased from EurisoTop. Dry dichloromethane (DCM) was collected from a MBraun SP800 purification system. The remaining solvents were HPLC graded. Moisture sensitive reaction was performed in flame‐dried glassware under positive pressure of N_2_. Analytical thin layer chromatography was performed using pre‐coated aluminum‐packed plates (Merck Silica gel 60 F_254_) and visualized under UV irradiation (254 or 365 nm) and/or dipping into KMnO_4_‐ or Ninhydrin stain followed by gently heating. Flash column chromatography was carried out using silica gel—high purity grade (w/Ca, ≈0.1%, 230–400 mesh particle size, 60 Å pore size) as the stationary phase acquired by Sigma Aldrich. Ultraviolet‐visible (UV–vis) absorbance spectra were measured using a Thermo Scientific Nanodrop 2000c.

Nuclear magnetic resonance (NMR) spectra were recorded on a Varian Mercury 400 MHz spectrometer and the spectra were recorded either as ^1^H‐NMR 400 MHz or ^13^C‐NMR 101 MHz. All spectra were referenced to the solvent peak; the chemical shifts were reported in ppm relative to the residual solvent peak. Analysis of the spectra was performed using MestReNova. The following abbreviations were used to indicate the multiplicity in the NMR spectra: s = singlet, d = doublet, t = triplet, q = quartet, dd = double doublet, m = multiplet. Coupling constants were reported in hertz (Hz) as the mean value between coupled hydrogen atoms. ^13^C‐NMR spectra were acquired in a broadband decoupled mode. High‐resolution mass spectrometry (HR‐MS) was recorded on a Bruker Maxis Impact time‐of‐flight mass spectroscopy (TOF‐MS) using electrospray ionization (ESI+). The spectra were calibrated to an internal standard and analyzed with the DataAnalysis software.

MALDI measurements were recorded using a Bruker AutoFlex II MS with nitrogen laser (337 nm) and 20 kV accelerating voltage with a grid voltage of 90%. At least 100 laser shots covering the complete spot were accumulated for each spectrum. For drug‐to‐antibody ratio of the prepared conjugates, sinapinic acid (20 mg mL^−1^) in 50% acetonitrile with 0.1% trifluoroacetic acid was used as matrix. Antibody solution (1 mg mL^−1^) was mixed with an equal volume of matrix, and 2 µL of the resulting mixture was loaded onto MTP 384 ground steel target plate. The extent of modification was determined by subtracting the ADCs’ *m*/*z* values from the native antibody *m*/*z* and dividing by the molecular weight of mitomycin linker (542 g mol^−1^).

Analytical HPLC analysis was carried out on an Agilent 1260 Infinity II with an Agilent ZORBAX eclipse Plus C18 column with a particle size of 3.5 𝜇m, a length at 150 mm and an internal diameter of 4.6 mm. Mobile phases were ultrapure water (MQ) and acetonitrile that was cooled overnight in the fridge to 4 °C and kept on ice during the HPLC analysis. MQ was received from Milli Q direct 8 system (Millipore). Furthermore, all the samples were kept on ice (0 °C) until analysis to avoid side‐degradation reactions.

### Synthesis of Compound **1b**

2‐hydoxyethyldisulfide (300 mg, 1.94 mmol, 1 equiv.) was dissolved in CH_2_Cl_2_ (16 mL) and subsequently the addition of 4‐nitrophenyl chloroformate (871 mg, 4.32 mmol, 2.23 equiv.). The mixture was bubbled through with N_2_ and cooled to 0 °C followed by dropwise addition of TEA (1.08 mL, 7.76 mmol, 4 equiv.). After addition, the reaction was left to heat to room temperature and stirred for two h. The reaction was quenched with NH_4_Cl, washed with brine, dried over Na_2_SO_4_, and concentrated in vacuo. The crude was purified with flash column chromatography (1:1 pentane:CH_2_Cl_2_ to 3:7 pentane: CH_2_Cl_2_) yielding the pure product as a yellow powder (467 mg, 0,964 mmol, 50% and 61%). ^1^H NMR (400 MHz, Chloroform‐d) *δ* 8.28 (d, J = 9.2 Hz, 4H), 7.39 (d, J = 9.2 Hz, 4H), 4.57 (t, J = 6.5 Hz, 4H), 3.08 (t, J = 6.5 Hz, 4H). ^13^C NMR (101 MHz, Chloroform‐*d*) *δ* 155.47, 152.48, 145.66, 125.50, 121.91, 66.89, 36.90. HRMS (ESI+) *m/z* calculated (calcd.) for C_18_H_16_N_2_O_10_S_2_ + Na^+^: 507.0139; found: 507.0173, calcd. for C_18_H_16_N_2_O_10_S_2_ + K^+^: 522.9978; found: 522.9924, calcd. for 2C_18_H_16_N_2_O_10_S_2_ + Na^+^:991.0384; found: 991.047

### Synthesis of Compound **1**

**1b** (22.2 mg, 0.0458 mmol, 2.4 equiv.) was dissolved in dry DMF (0.5 mL) and stirred under N_2_. In another flask mitomycin C (6.4 mg, 0.01914 mmol, 1 equiv.) and TEA (3.99 µL, 0.0287 mmol, 1.5 equiv.) was dissolved in dry DMF (0.25 mL) followed by dropwise addition to the stirring reaction mixture. HOBt (17.2 mg, 0.127, 6.7 equiv.) was subsequently added and the reaction was left in dark for 24 h, concentrated and purified by flash column chromatography (1:1 pentane:EtOAc to 3:7 pentane:EtOAc) to yield the product as a purple powder (7.4 mg, 0.011 mmol, 57%). R_f_ (3:7 Pentane:EtOAc) = 0.26. ^1^H NMR (400 MHz, Chloroform‐*d*) *δ* 8.29 (d, *J* = 9.2 Hz, 2H), 7.40 (d, *J* = 9.2 Hz, 2H), 4.84 (dd, *J* = 10.8, 4.7 Hz, 1H), 4.53 (t, *J* = 6.6 Hz, 2H), 4.39–4.26 (m, 3H), 3.68 (dd, *J* = 11.1, 4.7 Hz, 1H), 3.49 (dd, *J* = 13.4, 1.9 Hz, 1H), 3.44 (d, *J* = 4.6 Hz, 1H), 3.33 (dd, *J* = 4.6, 1.8 Hz, 1H), 3.20 (s, 3H), 3.03 (t, *J* = 6.6 Hz, 2H), 2.96 (t, *J* = 6.7 Hz, 2H), 2.89 (t, *J* = 5.7 Hz, 1H), 1.76 (s, 3H). ^13^C NMR (101 MHz, CDCl_3_) *δ* 160.74, 156.42, 155.51, 154.43, 152.51, 147.09, 125.52, 121.99, 110.57, 105.58, 105.41, 66.98, 64.74, 62.43, 60.51, 49.94, 48.79, 43.70, 41.97, 41.37, 40.13, 36.80, 36.76, 14.37, 8.05. HRMS (ESI+) *m/z* calculated (calcd.) for C_27_H_29_N_5_O_12_S_2_ + H^+^: 680.1328, found: 680.1328; C_27_H_29_N_5_O_12_S_2_ + Na^+^: 702.1146, found: 702.1149; C_27_H_29_N_5_O_12_S_2_ + K^+^: 718.0886, found: 718.0891.

### Synthesis of Compound **2b**

3‐mercapto‐3‐methylbutan‐1‐ol (200 mg, 1.66 mmol, 1 equiv.) was dissolved in CH_2_Cl_2_:EtOAc (1:1, 52 mL in total). CuCl_2_ (1.120 g, 8.33 mmol, 5 equiv.) was dissolved in 0.5 m HCl (36 mL) followed by addition to the organic phase. The reaction was left stirring for 1.5 h at r.t. followed by extraction of the reaction mixture with CH_2_Cl_2_ and afterward of the aqueous phase. The organic phases were combined, washed with brine, dried with Na_2_SO_4_, filtered, and concentrated yielding the pure product as a clear oil (289.3 mg, 1.212 mmol, 73%). ^1^H NMR (400 MHz, Chloroform‐*d*) *δ* 3.81 (t, *J* = 7.1 Hz, 4H), 1.86 (t, *J* = 7.1 Hz, 5H), 1.32 (s, 13H). ^13^C NMR (101 MHz, Chloroform‐*d*) *δ* 59.85, 48.55, 44.14, 28.82. HRMS (ESI+) *m/z* calculated (calcd.) for C_10_H_22_O_2_S_2_ + Na^+^: 261.0953, found: 261.0950; Cald. for C_10_H_22_O_2_S_2_ + 2Na^+^‐H^+^: 283.0772, found: 283.0708

### Synthesis of Compound **2c**

**2b** (86.7 mg, 0.364 mmol, 1 equiv.) was dissolved in dry CH_2_Cl_2_ (0.75 mL) followed by addition of TEA (125 µL, 0.895 mmol, 2.5 equiv.). In another flask 4‐nitrophenyl chloroformate (207 mg, 1.03 mmol, 2.82 equiv.) was dissolved in dry CH_2_Cl_2_ (0.75 mL), cooled to 0 °C, and the mixture containing TEA and 2‐hydroxytheyldisulfide was added dropwise to the stirring mixture. After addition, the ice was removed, and the reaction mixture was left stirring as a slurry at room temperature for three h. The reaction was diluted with CH_2_Cl_2_ (8 mL) and quenched with NH_4_Cl, washed five times with brine, and dried over Na_2_SO_4_. The crude was concentrated yielding the product as a pale yellow oil (142.1 mg, 0.25 mmol, 69%). ^1^H NMR (400 MHz, Chloroform‐*d*) *δ* 8.28 (d, *J* = 9.1 Hz, 4H), 7.38 (d, *J* = 9.1 Hz, 4H), 4.43 (t, *J* = 7.1 Hz, 4H), 2.05 (t, *J* = 7.1 Hz, 4H), 1.37 (s, 12H). ^13^C NMR (101 MHz, Chloroform‐*d*) *δ* 155.62, 152.55, 145.54, 125.46, 121.94, 66.67, 48.12, 40.24, 28.64. HRMS (ESI+) *m/z* calculated (calcd.) for C_24_H_28_N_2_O_10_S_2_ + Na^+^: 591.1077, found: 591.1083; Cald. for C_24_H_28_N_2_O_10_S_2_ + K^+^: 607.0817, found: 607.0885;

### Synthesis of Compound **2**

**2c** (38.6 mg, 0.0678 mmol, 2 equiv.) was dissolved in dry DMF (1 mL) and stirred under N_2_. In another flask mitomycin C (11.35 mg, 0.0339 mmol, 1 equiv.) and TEA (7.09 µL, 0.0508 mmol, 1.5 equiv.) was dissolved in dry DMF (0.5 mL) followed by dropwise addition to the stirring reaction mixture. HOBt (27.5 mg, 0.203 mmol, 6 equiv.) was subsequently added and the reaction was left in dark for 24 h, concentrated and purified by flash column chromatography (1:1 pentane:EtOAc to 3:7 pentane:EtOAc) to yield the product as a purple powder (13.43 mg, 0.0176 mmol, 52%). R_f_ (3:7 Pentane:EtOAc) = 0.31. ^1^H NMR (400 MHz, Chloroform‐*d*) *δ* 8.28 (d, *J* = 9.1 Hz, 2H), 7.38 (d, *J* = 9.2 Hz, 2H), 4.88 (dd, *J* = 10.8, 4.7 Hz, 1H), 4.48–4.36 (m, 3H), 4.31–4.15 (m, 3H), 3.67 (dd, *J* = 11.0, 4.7 Hz, 1H), 3.49 (dd, *J* = 13.4, 1.9 Hz, 1H), 3.42 (d, *J* = 4.6 Hz, 1H), 3.29 (dd, *J* = 4.6, 1.8 Hz, 1H), 3.19 (s, 3H), 2.01 (t, *J* = 7.1 Hz, 2H), 1.90 (t, *J* = 7.3 Hz, 2H), 1.76 (s, 3H), 1.34 (s, 6H), 1.29 (s, 6H). ^13^C NMR (101 MHz, CDCl_3_) *δ* 178.58, 176.05, 160.95, 156.36, 155.68, 154.42, 152.55, 147.15, 145.58, 125.46, 121.96, 110.71, 105.63, 105.35, 77.16, 66.73, 64.35, 62.31, 49.90, 48.85, 48.25, 48.01, 43.58, 42.12, 40.25, 40.14, 28.63, 28.62, 28.58, 8.03. HRMS (ESI+) *m/z* calculated (calcd.) for C_33_H_41_N_5_O_12_S_2_ + H^+^: 764.2266, found: 764.2288; Cald. for C_33_H_41_N_5_O_12_S_2_ + Na^+^: 786.2085, found: 786.2133;

### Fluorescence Labeling Antibodies

Anti *S. aureus* antibody (catalog nr.:PA1‐7246), anti‐FITC (catalog nr.: 31242), and Alexa Flour NHS ester 680 antibody were purchased from ThermoFisher. Protein concentrations were determined using UV–vis (*λ*_max_ = abs_280 nm_) with *ε*_protein_ = 210 000 m ^−1^ cm^−1^. Solutions of antibodies were buffer exchanged by the use of spin filters (Amicon spin filters, 30 kDa cutoff, regenerated cellulose) to 0.1 m sodium bicarbonate buffer, pH 8.3, and adjusted to a final protein concentration of >3 g L^−1^. The fluorophore (Alexa Flour 680 NHS ester or Alexa Flour 647 NHS ester) was dissolved in DMSO (10 g L^−1^ final concentration) immediately before use and added to an antibody solution in an excess of 8 molar equivalents. The reactions were left at room temperature for 1 h under mild shaking (800 rpm) in the dark. The solutions were then buffer exchanged to 10 mm PBS, pH 7.4 by the use of spin filters (Amicon spin filters, 30 kDa cutoff, regenerated cellulose) followed by purification by gel filtration through a NAP‐5 column (Sephadex G‐25 DNA grade).

### Synthesis of ADC (General Protocol)

Solutions of an antibody were buffer exchanged into a 10 mm PBS buffer, pH 7.4, using Amicon spin filters (MW cutoff 30 kDa, regenerated cellulose), with a final protein concentration at > 3 g L^−1^. Compound **1** (55 equiv. to protein) was added and the reaction was left shaking (800 rpm) in the dark at room temperature for 2 h. The ADC was purified with gel filtration through a NAP‐5 column (Sephadex, G‐25 DNA grade). The DAR was determined based on the distinct absorbance for mitomycin C at 360 nm and the absorbance at 280 nm for the protein.

### Drug Release from ADC

Triggered drug release was tested using solutions of GSH, DTT, NAC (each taken at 5 mm concentration), and BSA (0.76 µm) in PBS; unspecific drug release/linker stability was tested in PBS and in two different types of cell media, namely RPM1‐1640 medium containing 10% FBS, 1% P/S, 2 mm l‐Gln (denoted as FBS) and Modified M9 buffer (MM9) containing 1× M9 salts amended with 2 mm MgSO_4_, 0.1 mm CaCl. All the samples were incubated at 37 ^○^C, and time point (15 min, 1, 24 h) an aliquot was drawn out and analyzed on HPLC.

### Bacterial Strains and Growth Conditions

Bacterial cultures were stored in 15% glycerol at −80 °C. Single colonies were grown on brain heart infusion (BHI) agar and stored at 4–8 °C. Experiments were performed with overnight cultures. Each biological replicate was inoculated from a single colony in BHI broth at 37 °C, 180 rpm. For antibody binding and phagocytosis activity measured by flow cytometry, *S. aureus* Newman Δspa/Δsbi_mAm was used, which had low or no expression of staphylococcal protein A (SpA) and second immunoglobulin‐binding protein (Sbi) ref. [44], and contained a plasmid from which the fluorescent protein mAmetrine (mAm) was constitutively expressed.^[^ ^45^ ^]^ For antibody binding measured by microscopy, *S. aureus* ATCC29213 transformed with the plasmid pSB2019 was used,^[^ ^46^ ^]^ which expressed green fluorescent protein (GFP) constitutively. Plasmid maintenance was secured by growing and incubating cells in BHI media containing 10 µg mL^−1^ chloramphenicol. *S. aureus* ATCC 29213 was used for all other in vitro experiments. All experiments were performed with at least 3 biological replicates of independently grown overnight cultures.

### Media and Buffers

The growth media used in the experiments was brain heart infusion broth (BHI). Modified M9 buffer (MM9) contained M9 minimal salts 1 × (Sigma Aldrich M6030), 2 mm MgSO4, 0.1 mm CaCl2, 1 mm Thiamine‐HCL, 0.05 mm nicotinamide and 1 mL L^−1^ trace metals (TMS3). The pH of MM9 was adjusted to 7.4. After autoclavation components were sterile filtered and added to the buffer. MM9 media with carbon and casamino acids (MM9 + carbon) was supplemented with 1% glucose and 1% casamino acids. Assays involving MM9 with *N*‐acetyl cysteine (NAC) contained 5 mm NAC.

### MIC, MBC, and MBEC of Mitomycin C

A twofold serial dilution of mitomycin C (from 0.00625–3.2 mg L^−1^) was prepared in 96‐well plates in MM9 buffer, MM9 buffer with 5 mm NAC, MM9 media (MM9 buffer with 1% glucose and 1% casamino acids), or BHI. Overnight cultures were harvested by centrifugation (13 150 × *g* rpm, 8 min) and resuspended to OD_600_ of 0.05 in media matching the microtiter plates and inoculated into the plates by tenfold dilution, and incubated at 37 °C for 19 h 180 rpm before measuring OD_600_ (BioTek, Powerwave XS2). MIC was defined as the lowest concentration resulting in >90% inhibition of growth. MBC was determined by spotting out 10 µL from wells with no apparent growth on BHI agar and incubating for 24 h at 37 °C. MBC was determined as the lowest concentration resulting in no detection of viable cells (corresponding to a >99,9% reduction in CFU).

To determine MBEC, biofilms were grown on peg‐lids (Nunc 445497 Immuno TSP Lids) by inoculating the pegs in an overnight culture for 30 min at 37 °C and transferring to BHI for biofilm growth at 37 °C for 24 h with 120 rpm shaking. MBEC was determined in MM9 buffer with and without 5 mm NAC, MM9 media, and BHI. A twofold serial dilution of mitomycin C was prepared for these media (concentration range 128–0.25 mg L^−1^), and peg lids with biofilms incubated in these plates for 24 h at 37 °C and 120 rpm. After treatment, biofilms were washed by dipping peg lids twice in 96‐well plates with BHI to remove mitomycin C, and peg lids were then placed in a recovery plate with BHI, sonicated for 10 min (USCS1700 T, VWR, Westchester, PA, USA) to remove the biofilm from the peg‐lids, and incubated for 72 h at 37 °C, 50 rpm. Growth in the recovery plate was detected by measuring OD_600_ and the MBEC was determined as the lowest concentration resulting in no growth.

### MIC and MBC of Mitomycin C against Other Pathogens

*Escherichia coli* ATCC 8739, *Staphylococcus epidermidis* 1457, *P. aeruginosa* PA01, *Enterococcus faecalis* ATCC 7008, and Methicillin‐resistant *S. aureus* USA300 were grown overnight in BHI at 37 °C in Erlenmeyer flasks at 120 rpm and diluted to OD_600_ = 0.05 in MM9 minimal media. Twofold dilutions of mitomycin C in MM9 minimal media were prepared in 96‐well flat bottom plates to yield a concentration range of 0.03–8 mg L^−1^. Bacteria were then added to the wells (OD_600_ = 0.005), and the 96‐well plate was incubated at 37 °C, 100 rpm in darkness for 18–20 h. MIC and MBC were determined as described above.

### Antibody Binding to *S. aureus* Detected by Microscopy

Planktonic overnight cultures of *S. aureus* ATCC29213_GFP were immobilized by adsorption to SuperFrost Ultra Plus Adhesion Slides, blocked with 1% BSA, rinsed 3× with MM9 buffer (ref. [47]). *S. aureus*‐Alexa Fluor 647 (100 µL, 5 mg L^−1^) or anti‐FitC‐Alexa Fluor 647 (100 µL, 5 mg L^−1^) was added and incubated for 30 min at room temperature followed by gentle washing with MM9 buffer. Unstained negative controls were incubated with MM9 buffer. Bacteria were visualized by confocal laser scanning microscopy (CLSM, Zeiss LSM700) equipped with a 63x/NA1.4 Plan‐Apochromat objective using 488 and 639 nm excitation.

*S. aureus* biofilms were formed by inoculation of overnight culture into 96 well plates (IBIDI, 89626) for 2 h at 37 °C. Non‐adhered cells were then removed by rinsing, and wells were filled with BHI broth and incubated for 24 h at 37 °C. Biofilms were then washed, blocked, and hybridized with antibodies, Biofilm embedded cells were stained with SYTO41 and visualized by CLSM using 405 and 639 nm excitation.

### Binding of ADC vs Parent Antibody to *S. aureus* Detected by Flow Cytometry

Single colonies of *S. aureus* from tryptic soy broth (TSB) agar plates were grown in TSB with 10 ug mL^−1^ of chloramphenicol for 19 h at 37 °C, 180 rpm. Bacteria were diluted to OD600 = 0.05 in TSB with chloramphenicol, and grown at room temperature to midlog phase (OD600 = 0.427 corresponding to 4.27  × 10^8^ CFU mL^−1^) followed by wash and resuspension in RPMI‐H medium (Roswell Park Memorial Institute medium (RPMI), added 0.05% HSA) and stored at −20 °C. Freezer cultures were thawed at room temperature and diluted in RPMI‐H to 3.75 × 10^7^ CFU mL^−1^ before the experiments.

*S. aureus* was added to 96‐well plates (20 µL well^−1^) and mixed with 20 µL of ADC or antibody (*S. aureus* polyclonal rabbit IgG, Thermo Fisher) in threefold dilution series for 30 min at 4 °C, 600 rpm. Wells were washed with 160 µL of RPMI‐H at 2823 × *g* for 7 min. Goat‐anti‐rabbit‐IgG‐APC detection antibody (1 µg mL^−1^) (Molecular Probes, 199312) with 30 µL RPMI‐H was added to the bacteria and incubated for 30 min at 4 °C, 600 rpm. Unbound detection antibody was removed by washing and bacteria fixated with 100 µL of 1% paraformaldehyde (PFA) (Polysciences) in RPMI‐H. Quantification of ADC and antibody bacterial binding was measured with flow cytometry (BD FACSVerse) and data were analyzed with FlowJo Software (Version 10.8.1).

### Phagocytosis of Bacteria Opsonized by ADC and Parent Antibodies

Neutrophils were isolated from healthy blood donors using the Ficoll–Histopaque method.^[^ ^48^ ^]^ For opsonization, *S. aureus* was added to 96‐well plates (20 µL well^−1^) and incubated with 10 µL of 1% IgG/IgM depleted human pooled serum^[^ ^29^ ^]^ and 10 µL ADC, specific or non‐specific antibody in threefold dilution series for 15 min at 37 °C, 750 rpm. 10 µL of neutrophils (7.5 × 10^6^ cells mL^−1^) were added and incubated 15 min at 37 °C, 750 rpm. Phagocytosis was stopped and cells fixated by adding 80 µL 1.62% cold PFA to each well. Quantification of phagocytosis was done using flow cytometry and data was analyzed with FlowJo Software. The gating strategy used was based on neutrophil population gated on FSC‐SSC and GeoMFI of neutrophils using the mAmetrine channel.

### Antimicrobial Effect of ADC against Planktonic *S. aureus*

Overnight cultures were harvested by centrifugation, resuspended in MM9, and diluted to OD_600_ = 0.005. 100 µL of each culture was added to Eppendorf tubes and centrifuged. The formed pellets were resuspended in 100 µL of MM9 buffer containing the chosen concentrations of specific ADC or non‐specific ADC, with or without 5 mm NAC. In experiments with lower OD_600_ than 0.05 the pellet was not resuspended but ten µL of bacterial suspension was transferred to 90 µL containing the chosen concentration of *S. aureus* specific or non‐specific ADC. Blanks (without bacteria) and growth controls (with bacteria) contained MM9 media. All samples were incubated for 2 h at 37 °C, 180 rpm and centrifuged twice for 8 min, 1315 × *g*, and resuspended in MM9. Tenfold dilutions were made in MM9 of each sample, followed by plating 10 µL on agar and incubation for 19 h at 37 °C and then CFU enumeration. The antimicrobial effect was evaluated by comparing the CFU of treated samples with the CFU of the growth control from the same experiment.

### Antimicrobial Efficacy of Specific ADC against Biofilm Associated *S. aureus*

Biofilms were grown on peglids by inoculating them in an overnight culture (OD_600_ = 1) for 1 h at 37 °C, 120 rpm and then transferred to 96‐well plates containing BHI and inoculated for48 h at 37 °C, 120 rpm. Media was exchanged after 24 h. The peg‐lids were treated in 96‐well plates with twofold dilutions of specific or non‐specific ADC (0.5–2 mg mL^−1^) and incubated 15 min at 37 °C. 120 rpm. Unbound ADC was removed by incubation in MM9 for 2 h. Peglids were transferred to a microtiter plate with MM9 and sonicated for 10 min to remove biofilm. Tenfold dilutions were made from each treatment and spotted onto BHI agar plates followed by incubation at 37 °C before CFU enumeration was performed.

### Thiol Labeling of Planktonic *S. aureus*

Overnight cultures were washed twice (13 150 × *g*, 10 min) and resuspended inMM9 and diluted to OD_600_ = 1. Fluorescein maleimide (sample) or fluorescein (control) was added to the cultures for a final concentration of 0.1 mm. Bacteria were stained with SYTO60 (0.02 mm) and incubated for 30 min in the dark at 37 °C, 50 rpm. Stained bacteria were washed (14 500 rpm, 10 min) and resuspended in PBS. 20 µL of sample and control was added to a superfrost ultra plus slide for 10 min. Bacteria were then visualized with CLSM through a 100 × NA1.4 Zeiss Plan‐Aprochromat oil objective, using 488 and 639 nm wavelengths for excitation and emission, respectively.

### Thiol Labeling of *S. aureus* Biofilm

BHI supplemented with 5% plasma (100 µL) was incubated in IBIDI 96 well black µ‐Plate (IBIDI 89621) for 30 min. Wells were washed twice with fresh BHI and 200 µL of OD‐adjusted *S. aureus* overnight culture (OD_600_ = 1) was added and incubated for 2 h at 37 °C. Media was replaced with fresh BHI and incubated for 24 h at 37 °C 50 rpm. BHI was removed and replaced with 1% blocking agent (BSA in MM9) and incubated for 1 h. Wells were washed three times in MM9, and 99 µL of MM9 was added. 1 µL fluorescein maleimide (0.1 mm) or fluorescein (0.1 mm) was added to the wells, and incubated 30 min in the dark at 37 °C, 50 rpm. Subsequently, wells were washed three times with PBS and resuspended in 1× PBS (42 µL), and 8 µL SYTO60 (0.04 mm). Imaging was performed as described above.

### In Vitro Toxicity Test

The MOLT‐4 cells, a human T lymphoblast cell line with acute lymphoblastic leukemia were grown as a suspension culture in pre‐heated (37 °C) RPMI‐1640 Medium (Sigma R0883) containing 10% fetal bovine serum (Sigma F724), 2 mm l‐glutamine (Sigma G2150) and 1% penicillin/streptomycin (Sigma P0781). The suspended cells were grown in 75 cm^2^ culture flasks and stored up‐right in an incubator at 37 °C with 5% CO_2_.

Cell counting was carried out using a 1:1 ratio of Trypan Blue staining solution (Thermofisher) to cells and counting was fulfilled using an automated cell counter LUNA (Logos Biosystems*)*. Cells were in same procedure controlled for viability, which was always higher than 80%.

### Toxicity Test of ADC and Mitomycin C

MOLT‐4 cells in passage P9‐P11 (2.5 × 10^5^ cells mL^−1^) were seeded in a 96‐well plate followed by addition of a dilution series of either specific ADC or free mitomycin C (DMSO content were equalized). Control samples without specific ADC or free mitomycin C were also prepared with equivalent DMSO percentage. The samples were incubated with specific ADC or free mitomycin C for all 72 h at 37 °C, 5% CO_2_. After this, cell viability was assessed using PrestoBlue cell viability reagent (Thermofisher A13262) using a BioTek Synergy H1 microplate reader (*λ*_ex_/*λ*_em_ = 536 nm/619 nm). The experiment was reproduced three independent times with 3 replicates each time.

### Study Animals

8–10 week old C57bl/6j mice (Janvier Labs, Le Genest‐Saint‐Isle, France) were housed at The Animal Facilities Arhus University at standard room temperature, 12 h day/night cycle with water and food ad lib. The animals had an acclimatization period of 1 week prior to surgery. The study was approved by the Danish Animal Experiments Inspectorate under permission 2016‐15‐0201‐01121 and done under the supervision of the faculty vets.

### Inoculation of Steel Implants

The isolate *S. aureus* SAU060112 was used, a clinical isolate from a patient with a prosthetic joint infection^31^. Cultures from tryptic soy broth (TSB) agar plates were grown overnight in TSB media for 18 h at 37 °C, 180 rpm. The overnight culture was diluted in TSB to OD600 = 0.1 corresponding to 5 × 10^6^ CFU mL^−1^, and was added to a 50 mL falcon tube with the steel implants (Ento Sphinx, Pardubice IV, Czech Republic) and grown for 18 h at 37 °C. The implants were then transferred to fresh PBS before transportation to the animal facilities.

### Implant‐Associated Osteomyelitis Model

The surgical procedure was based on a model by Jørgensen et al.^[^ ^49^ ^]^ Briefly, mice were anesthetized with inhalation isoflurane (4–5% induction, 2% maintenance). Each animal was given an injection of buprenorphine 0.15 mg kg^−1^ s.c and the right hind leg was shaved. Implants were then surgically implanted transcortically through the left tibia at the proximal epiphysis. The implant was bent in a U shape and cut close to the skin on both sides. Afterward, the skin and adjacent tissue were manipulated to cover the implant and animals were returned to their cages. Buprenorphine 0.009 mg mL^−1^ was administered in the water the first 4 days post‐surgery.

### Deposition of Antibodies at Infection Site

3 days after infection animals were equally divided in to two groups by letter randomization. Group 1 (n = 7 infected implant, n = 3 sterile implant) was injected i.v. with antibody(*S. aureus* polyclonal rabbit IgG) conjugated to Alexaflour680. Group 2 (n = 7 infected implant, n = 3 sterile implant) was injected i.v.with unspecific antibody (FITC mice monoclonal IgG1, Thermo Fisher) conjugated to Alexaflour 680. The animals were scanned using in vivo imaging system (IVIS, Xenogen, Alameda, CA, USA) at baseline and 1, 4, and 24 h after injection. Fluorescent intensity from both legs of each animal was measured at excitation 675 nm, emission 720 nm. Analysis of data was done with Living Image Acquisition/Analysis Software Package. Fluorescence signal was counted by selecting the region of interest (ROI) of the implanted leg (ROI‐1) and the non‐implanted leg (ROI‐2) of each animal. The Average radiant efficiency ([p s^−1^ cm^−^^2^ sr^−1^]/[µW cm^−^^2^]) of the ROI of each animal was then calculated for both legs and ROI‐1 was subtracted from ROI‐2 to remove background noise (Figure 5C).

### In Vivo Efficacy of Antibacterial Treatment

7 days after infection, 14 mice with an infected implant from the in vivo imaging were randomly divided into three treatment groups; 1) NaCl 0.9%, 0.5 mL 24 h/s.c (n = 3), 2) Vancomycin (Bactocin, MIP Pharma GmbH) 110 mg kg^−1^/12 h/s.c (n = 5), 3) Vancomycin 110 mg kg^−1^/12 h/s.c + specific ADC 5 mg kg^−1^/24 h/i.v. (n = 5). The final concentration of ADC was 5 mg kg^−1^ of which 4.916 mg kg^−1^ was antibody and 86 µg kg^−1^ was mitomycin C. Animals were treated for 3 days followed by a 36 h antibiotic washout period to avoid carry‐over effect, and then euthanized by cervical dislocation. The tibia was exposed by surgical incision and the bone with implant was removed and snap‐frozen at −80 °C.

### Quantification of Bacterial Load

Implants were carefully removed from the tibial bone and then submerged in 1 mL PBS (11 mm, pH 7.5), vortexed for 30 s, and sonicated for 5 min at 45 kHz and 110 W (USCS1700 T, VWR). The tubes were vortexed 30 s again and sonicate from each sample was serially diluted in triplicates, plated onto 5% blood agar plates, and incubated at 37 °C for 48 h. CFU was enumerated and bacterial load was calculated.

### Comparison of ADC with Drug Combination Therapy

Implants were inoculated as previously described, but with minor optimizations. Implants were inoculated in falcon tubes with five implants in each tube instead of all implants in one tube and then kept in the overnight culture until implantation instead of transferring them to PBS after inoculation. Animals were operated following implant‐associated osteomyelitis model. 7 days after infection, 40 animals were randomly divided into four treatment groups; 1) NaCl 0.9%, 0.5 mL 24 h/s.c (n = 10), 2) Vancomycin 110 mg kg^−1^/12 h/s.c (n = 10), 3) specific ADC 5 mg kg^−1^/24 h/i.v. (n = 10), 4) Vancomycin 110 mg kg^−1^/12 h/s.c + specific ADC 5 mg kg^−1^/24 h/i.v. (n = 10). Following 3 days of treatment and 36 h of antibiotic washout, animals were then euthanized and bone with implant was removed and bacterial load was analyzed immediately after, following the quantification of bacterial load protocol.

### Quantification of Treatment Efficacy of ADC Components

The experiment was carried out similarly for the comparison of ADC with drug combination therapy study, with the same optimizations but with the following treatment groups: 1) NaCl 0.9%, 0.5 mL 24 h/s.c (n = 10), 2) specific ADC 5 mg kg^−1^/24 h/i.v. (n = 10), 3) mitomycin C 86 µg kg^−1^/24 h/i.v. (n = 10), 4) specific antibody 5 mg kg^−1^/24 h (n = 10), 5 non‐specific ADC 5 mg kg^−1^/24 h (n = 10).

### Data Treatment

All viability data were prepared by subtracting background from the raw data and normalizing it to the independent viability controls. The viability was then plotted as a function of the logarithm of the concentration. The independent IC_50_ values were estimated by fitting to a sigmoidal curve (four parameters, variable slope) using the software GraphPad Prism 9.

Statistical analysis was carried out via one‐ or two‐way ANOVA, based on at least 3 independent experiments, using GraphPad Prism. The sample size for each experiment, where appropriate, is noted in the respective figure caption.

## Conflict of Interest

The authors declare no conflict of interest.

## Author Contributions

A.T. and M.I.J. contributed equally to this work. S.C., L.Ø., N.P.J., R.L.M., and A.N.Z. contributed to conceptualization. A.T., M.I.J., S.C., N.P.J., R.L.M., and A.N.Z. contributed to methodology. A.T., M.I.J., L.N.G., D.M.I., A.B.K., S.C., S.S.M., A.B.S., E.F., and L.d.V. contributed to investigation. R.L.M. and A.N.Z. contributed to writing—original and revised drafts.

## Supporting information

Supporting Information

## Data Availability

The data that support the findings of this study are available from the corresponding author upon reasonable request.
