# Supplementary material for: Antibody‐Drug Conjugates to Treat Bacterial Biofilms via Targeting and Extracellular Drug Release
Source: Adv Sci (Weinh). 2023 Jun 8;10(23):2301340. doi: 10.1002/advs.202301340 (PMC10427384; doi:10.1002/advs.202301340)

ADVANCED SCIENCE Open Access

Supporting Information

for *Adv. Sci.*, DOI 10.1002/advs.202301340

Antibody-Drug Conjugates to Treat Bacterial Biofilms via Targeting and Extracellular Drug Release

Anne Tvilum, Mikkel I. Johansen, Lærke N. Glud, Diana M. Ivarsen, Amanda B. Khamas, Sheiliza Carmali, Snehit Satish Mhatre, Ane B. Søgaard, Emma Faddy, Lisanne de Vor, Suzan H. M. Rooijakkers, Lars Østergaard, Nis P. Jørgensen, Rikke L. Meyer* and Alexander N. Zelikin**

Supplementary Materials for

Antibody-drug conjugates to treat bacterial biofilms via targeting and extracellular drug release

Anne Tvilum, Mikkel I. Johansen et al.

*Corresponding authors:
Nis P. Jørgensen nisjoerg@rm.dk
Rikke L. Meyer rikke.meyer@inano.au.dk
Alexander N. Zelikin zelikin@chem.au.dk

This PDF file includes:

Supplementary Figure
NMR spectra for compounds

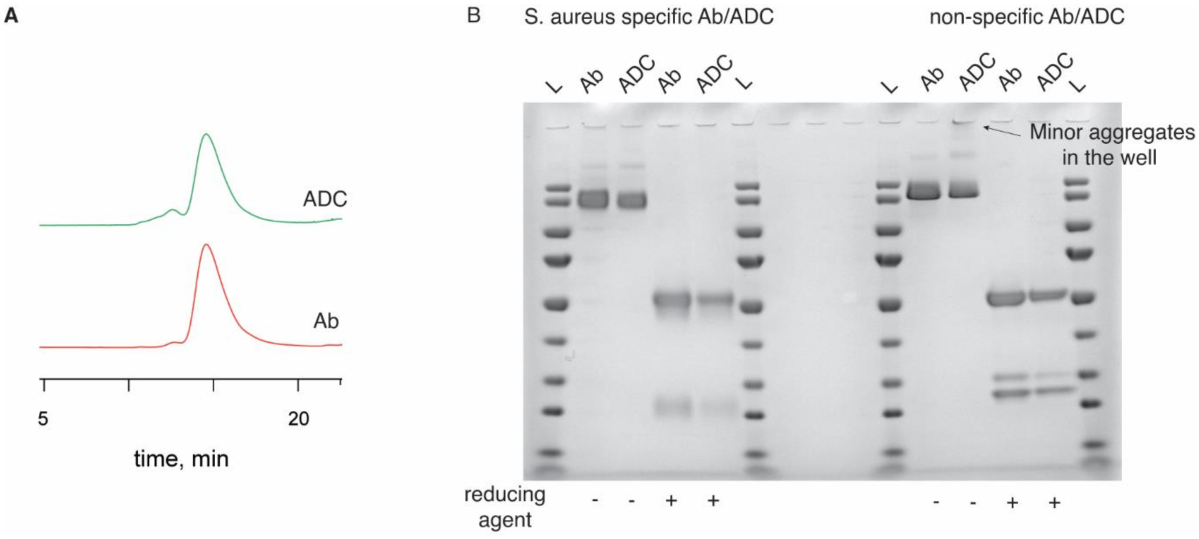

Supplementary Figure S1: Size exclusion chromatography (A) and gel electrophoresis (B) analyses of the antibodies and the antibody-drug conjugates derived thereof, demonstrating that ADC have minimal aggregation in solution upon storage. Drug-antibody ratio (DAR) for S.aureus specific ADC was 8, for the non-specific antibody was 13.

NMR spectra

NMR of 1b

^{1}H-NMR:

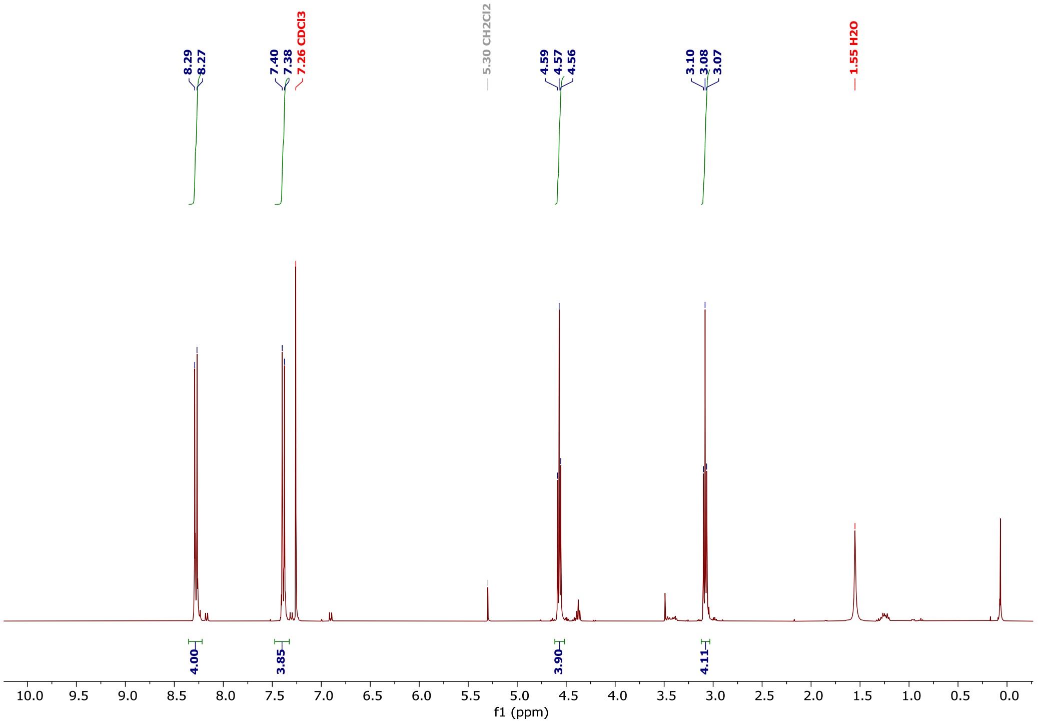

^{13}C-NMR:

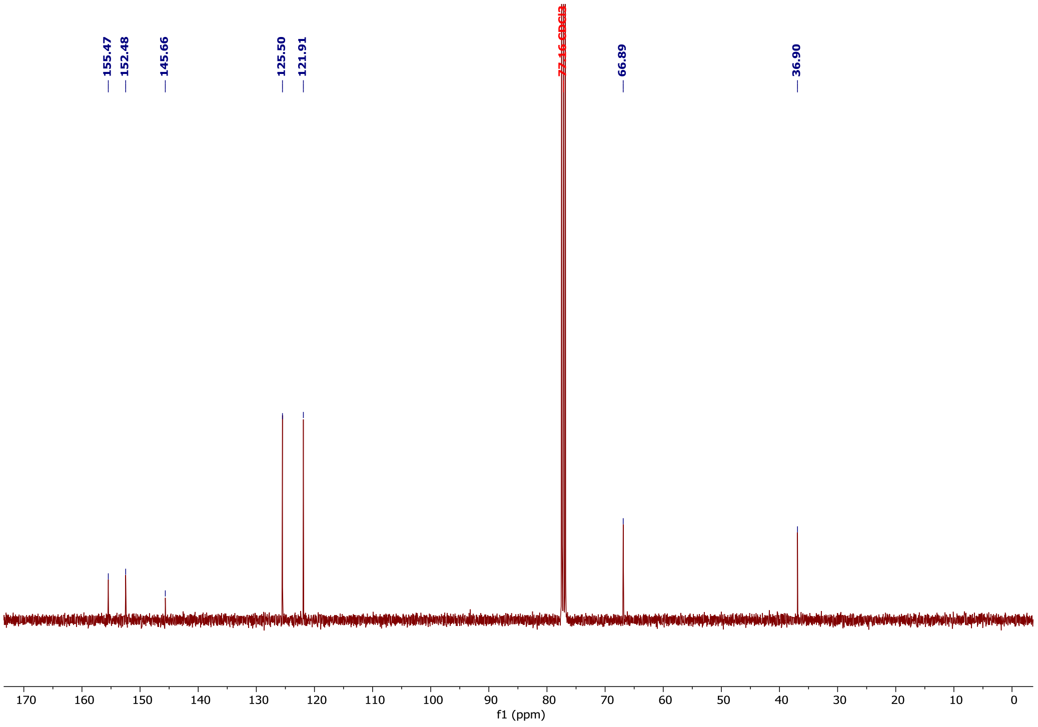

NMR of 1

^{1}H-NMR:

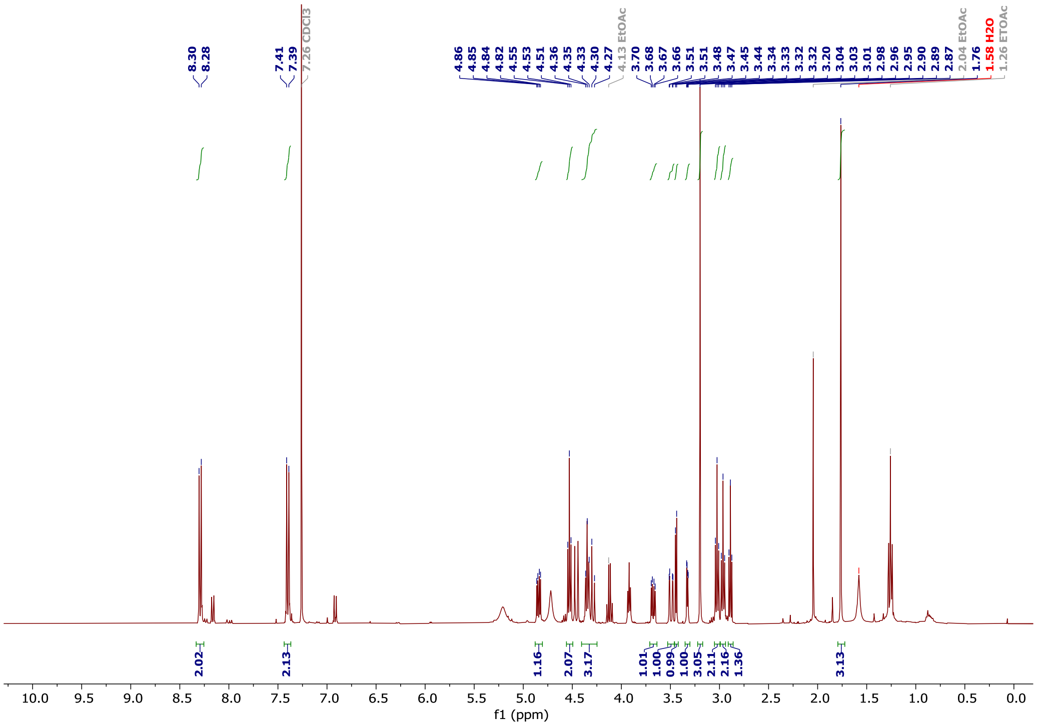

^{13}C-NMR:

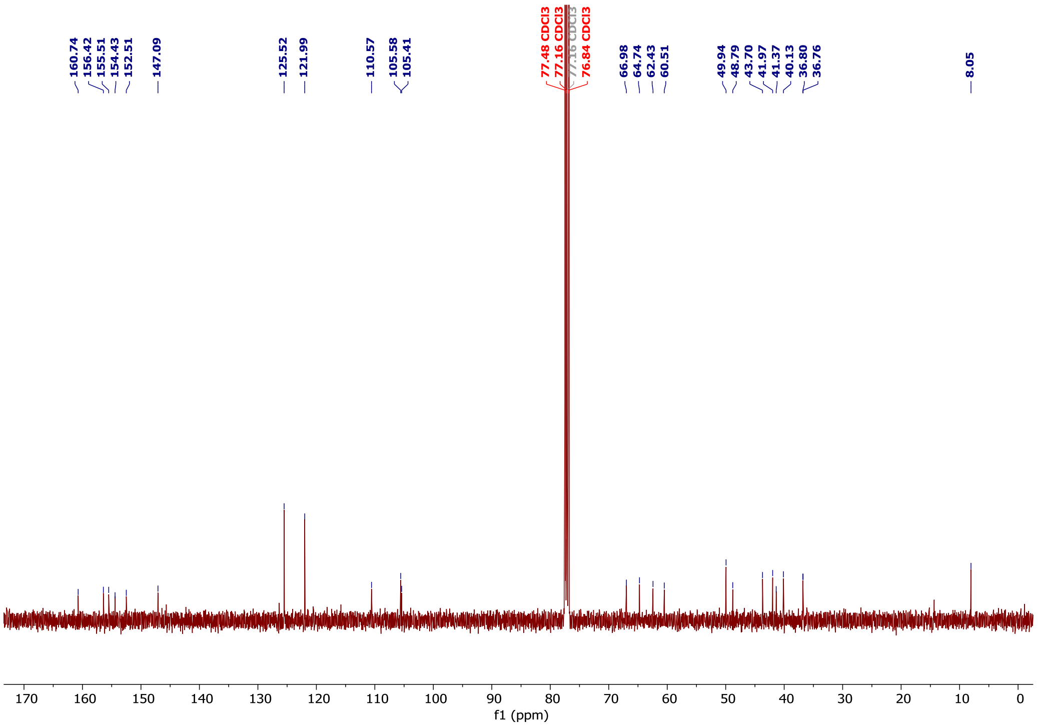

NMR of 2b

^{1}H-NMR:

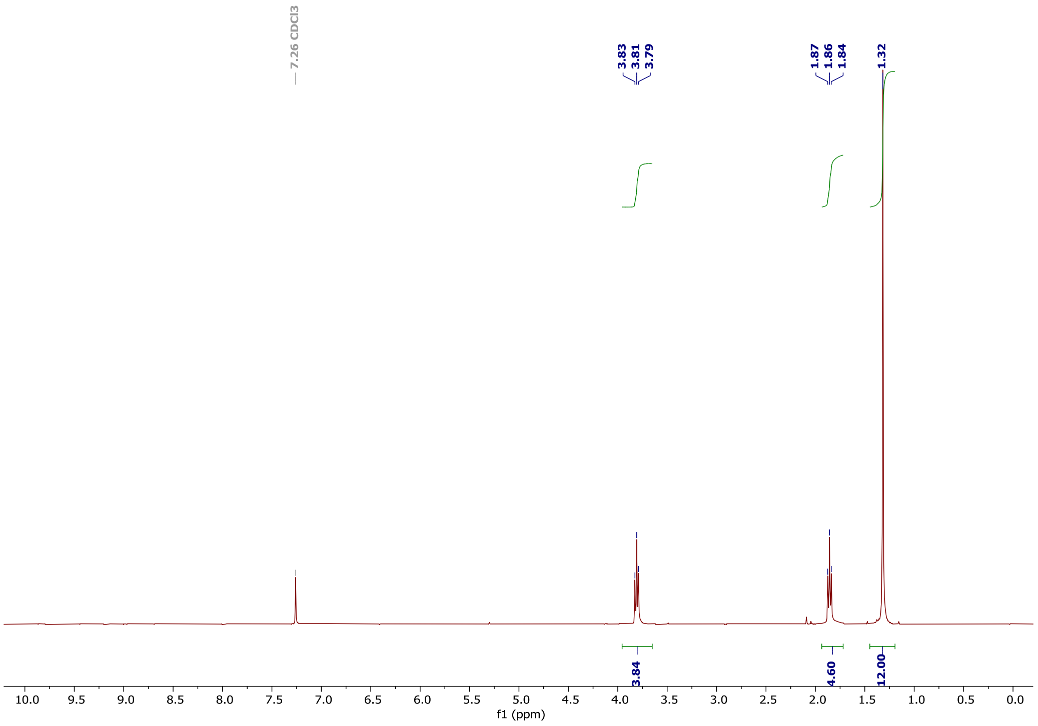

^{13}C-NMR:

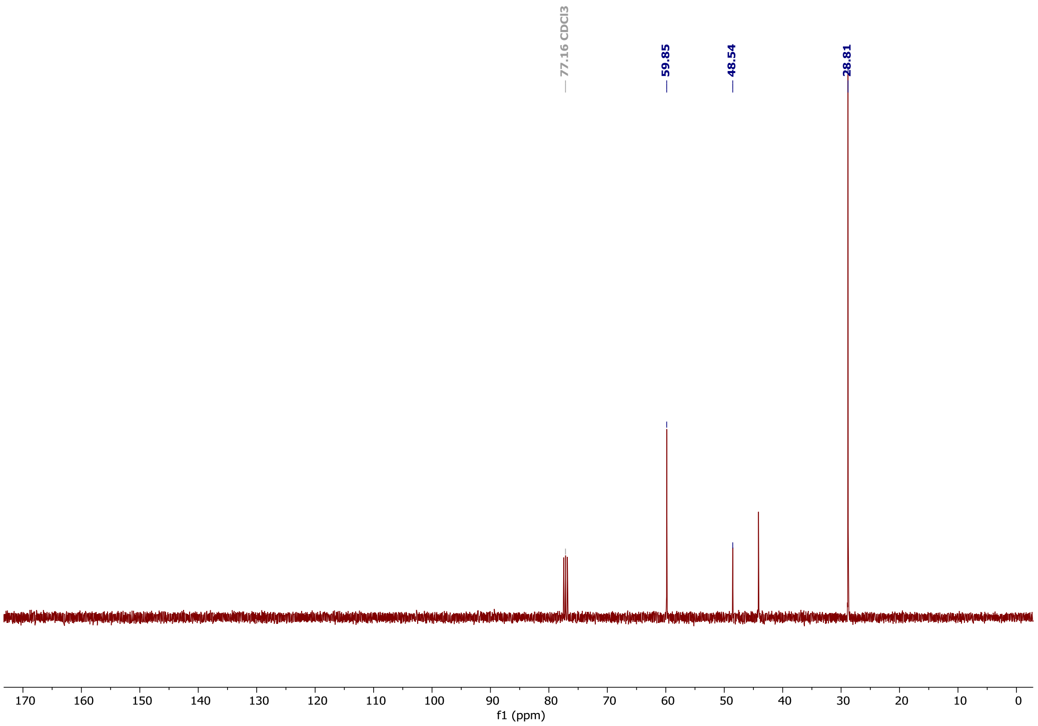

NMR of 2c

1H-NMR:

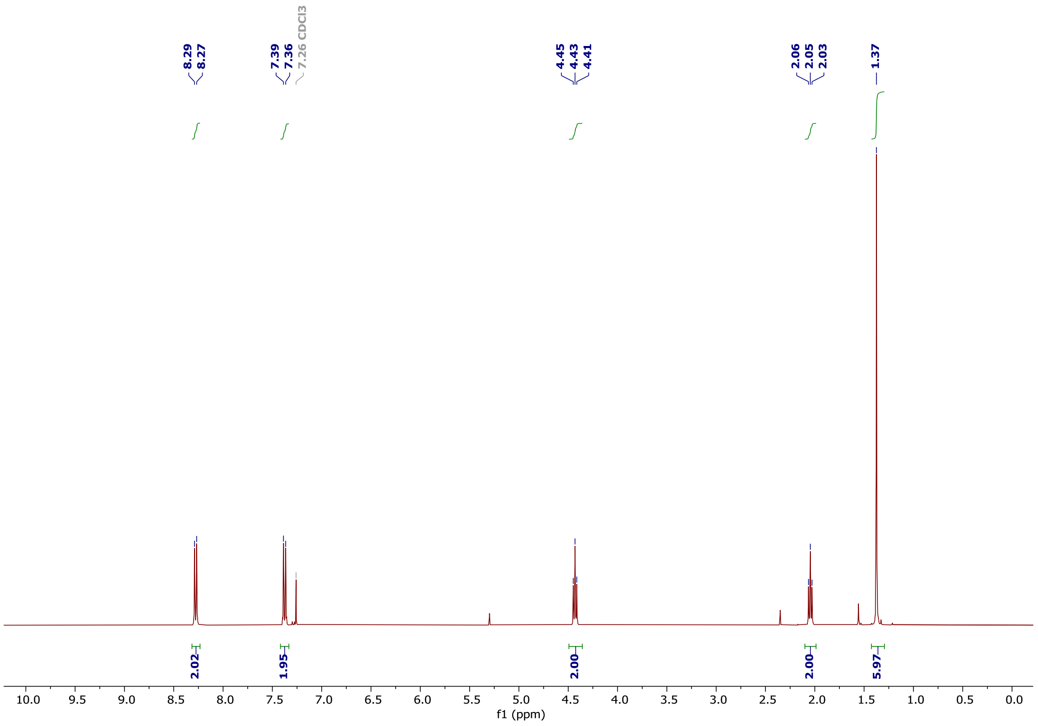

13C-NMR:

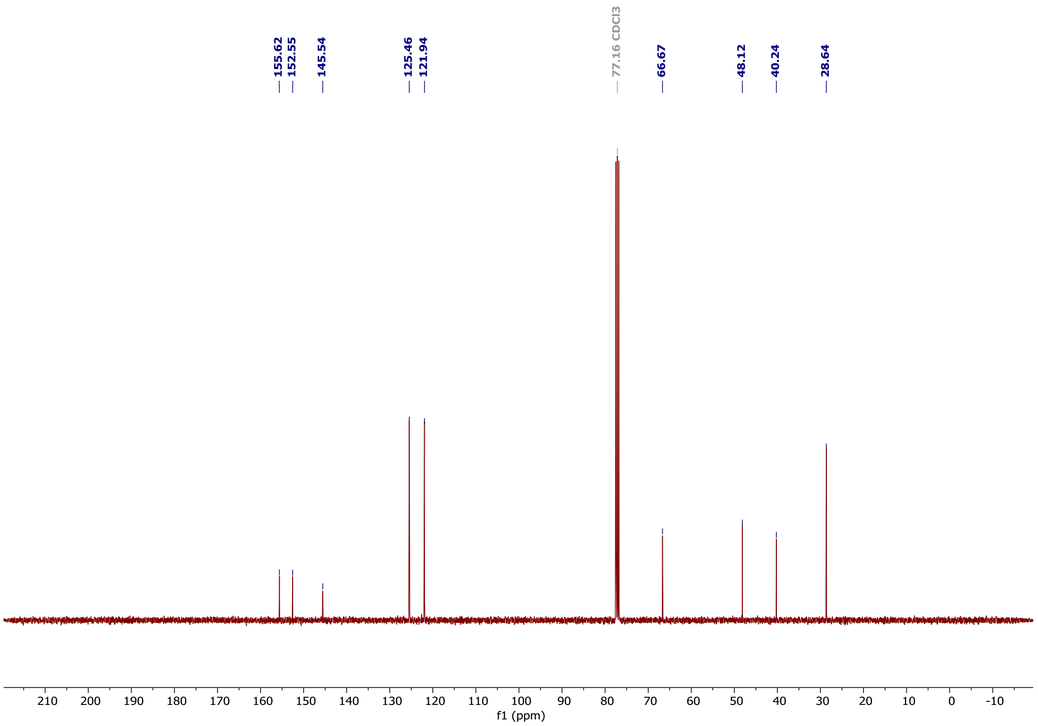

NMR of 2

^{1}H-NMR:

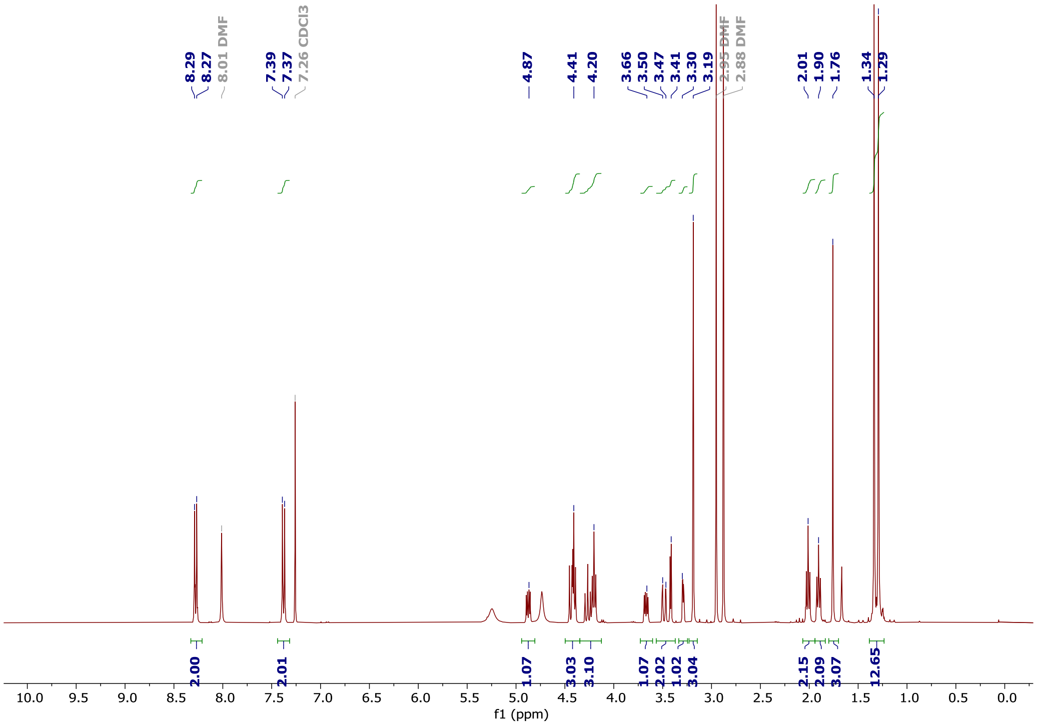

^{13}C-NMR:

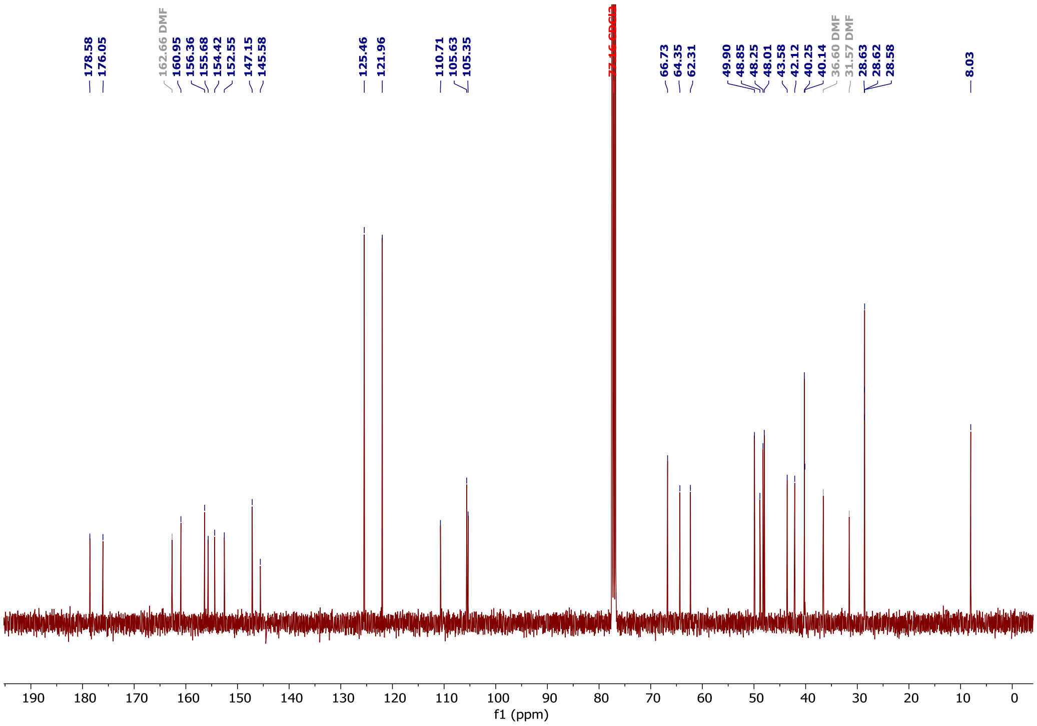

Supplement: Supplementary file 1 — Supporting Information [file ADVS-10-2301340-s001.pdf]
